# LOTUS: A low-cost time-lapse automated imaging system for spatio-temporal analysis of microbial colony or biofilm development

**DOI:** 10.1371/journal.pone.0339652

**Published:** 2026-01-23

**Authors:** Ryunosuke Sakai, Yifan Zhao, Martin Robert

**Affiliations:** Graduate School of Pharmaceutical Sciences, Kyoto University, Kyoto, Japan; SRMIST (Deemed to be University): SRM Institute of Science and Technology (Deemed to be University), INDIA

## Abstract

The proliferation of low-cost single-board computers and 3D printers has considerably accelerated open science. In the life sciences, for both research and educational purposes, there is a growing trend to develop affordable imaging systems rather than purchasing specialized commercial instruments. However, existing solutions often lack diversity of imaging modes or adequate throughput. To fill this gap, we developed LOTUS, a low-cost (~$550 USD) automated imaging system built from 3-D printed components that integrates motorized sample positioning with interchangeable light-emitting diodes (LED) sources and optical filters for spatio-temporal analysis of microbial colony or biofilm development. LOTUS images up to nine samples at fixed time intervals (e.g., 20 min) in four modes: bright-field transillumination (biomass), bright-field epi-illumination (morphology), and dual-color epi-fluorescence (gene expression or other types of reporter analysis). Validation experiments demonstrated stable and reproducible timing and positioning accuracy over 3 days and homogeneity of LED illumination and captured images enabling semi-quantitative analysis. We demonstrated LOTUS capabilities by imaging *E. coli* biofilms expressing fluorescent reporter proteins (GFPmut2 and mCherry) over 5 days and tracking fluorescence intensity dynamics following sub-MIC ampicillin treatment. LOTUS represents a versatile and cost-effective semi-quantitative platform for parallel monitoring of colony or biofilm development and fluorescent reporter expression pattern. This open-source system makes automated time-lapse live imaging accessible for research and educational applications.

## Introduction

Live imaging is essential for understanding the spatio-temporal dynamics of developmental processes by monitoring cellular events such as gene expression. Commercially available automated imaging solutions enable long-term monitoring of multiple samples [1–3] but come with high costs limiting accessibility. Even in shared facilities, extended multi-sample time-series experiments may not be possible. In addition, macro-scale applications only require moderate resolution (< 1 mm) and large fields of view (1–25 mm) rather than high-magnification but can be enhanced by automation and flexible illumination modes.

In this context, the open-science movement has been gaining momentum [4–9], enabled by the broad availability of low-cost single-board computers and cameras, 3D-printers, LEDs, as well as printed circuit boards (PCB) and control circuits [10–18]. These enable quick and simple prototyping of customized imaging hardware at low cost, for specific imaging purpose and sample formats, something often difficult with commercial solutions.

Several open-source imaging systems have been developed for various applications, including microbial colony and biofilm imaging. FlyPi allows high-quality bright-field or fluorescence imaging [5] of small model organisms but only for a single sample, limiting parallel experiments. Alternately, the Picroscope enables parallel micro-imaging by using multiple cameras [17] but is limited to bright-field illumination. For biofilm imaging one solution elegantly addressed the problem of condensation on lids during extended imaging of agar plates using robotic lid lifting [10]. However, the system only supports bright-field stereomicroscopy and repeated dish opening while maintaining high humidity in the system can lead to contamination. There are other interesting examples for spatio-temporal and high-throughput imaging of bacterial colonies [14,15]. However, these systems are limited to a single blue light excitation, which is not optimal for quantitative analysis using different fluorescent proteins and lack bright-field illumination. Another alternative enables fluorescence imaging of multiple wells but uses hybrid RGB LEDs for illumination without automated filter exchange limiting selectivity for applications with multiple fluorescent proteins [16,18]. Overall**,** while there are multiple useful solutions with individual capabilities, no single low-cost system integrates automated sample positioning, multiple excitation sources with filter switching for both bright-field and fluorescence imaging modes.

For comprehensive analysis of biofilm development and gene expression dynamics, multiple imaging modes are required: 1) bright-field epi-illumination to visualize surface morphology (wrinkles and rings) and guide omics sampling strategies, 2) transillumination for biomass quantification, 3) multi-color fluorescence with exchangeable excitation sources and filters for fluorescent reporter analysis such as green (GFP) or red fluorescent protein (RFP), and 4) automated multi-sample imaging over extended periods (days to weeks). While some of these features have been implemented, they have not been integrated in a single instrument.

Inspired by these projects we developed LOTUS (LOw-cost Time-lapse aUtomated imaging System), to fill this gap. The system automatically images up to nine samples in both bright-field and fluorescence for several days. Its key features include motorized two-axis (xy) sample positioning, exchangeable LED illumination modules and filter switching. LOTUS integrates the most desirable features of previously reported solutions and is suitable for macro-scale imaging (roughly 1 mm to 25 mm at a resolution below 0.14 mm) of developing microbial colonies or biofilms. Here, we describe LOTUS conceptualization, design, fabrication and assembly into an integrated multi-mode imaging system. We also validate and benchmark the system and offer a comparison with other existing systems while describing its limitations. Finally, we demonstrate its use to derive semi-quantitative time-series data about *E. coli* biofilm development and GFP expression level using fluorescent transcriptional reporters. Overall, LOTUS provides a reliable and accessible solution for biological imaging for both research and educational purposes.

## Materials and methods

### Experimental procedures

#### Bacterial strains.

All the *Escherichia coli* (*E. coli*) MG1655-derived strains carrying reporter constructs [19] and fluorescent protein plasmids used in this study are listed in Table 1.

**Table 1:** Strain and plasmid used in this study.

Experimental models: Organisms/strains		
Reagent or Resource	Source	Identifier
*E. coli* U66	[19]	*E. coli* MG1655 strain carrying pUA66 plasmid
*E. coli* rsd	[19]	*E. coli* MG1655 strain carrying pUA66-rsD (Inserted *rsd* promoter region to multicloning site of plasmid)
*E. coli* ribB	[19]	*E. coli* MG1655 strain carrying pUA66-ribB (Inserted *ribB* promoter region to multicloning site of plasmid)
*E. coli* cld	[19]	*E. coli* MG1655 strain carrying pUA66-cld (Inserted *cld* promoter region to multicloning site of plasmid)
*E. coli* ompA	[19]	*E. coli* MG1655 strain carrying pUA66-ompA (Inserted *ompA* promoter region to multicloning site of plasmid)
*E. coli* hipB	[19]	*E. coli* MG1655 strain carrying pUA66-hipB (Inserted *hipB* promoter region to multicloning site of plasmid)
*E. coli* U66p5mCherry	This study	U66 promoter strain carrying p5mCherry plasmid
*E. coli* rsd + p5mCherry	This study	rsd promoter strain carrying p5mCherry plasmid
**Recombinant DNA**		
p5mCherry	[20]	N/A

p5mCherry was developed by Brian Pfleger and obtained from Addgene (plasmid # 92023). The *E. coli* promoter collection [19] was obtained from Horizon Discovery: https://horizondiscovery.com/en/non-mammalian-research-tools/products/e-coli-promoter-collection

### Bacterial culture and biofilm production

Bacteria from glycerol stocks stored at 80°C were streaked onto Luria-Bertani (LB) agar plates (1% Bacto tryptone (BD Difco, Sparks, MD, USA), 0.5% Bacto yeast extract (BD Difco), 1% sodium chloride (Nacalai Tesque, Inc., Kyoto, Japan) and 1.5% Bacto Agar (BD Difco)), and incubated for 16 h at 37°C. Transformation was done using the calcium chloride method (S3 File). Biofilms were prepared according to Serra et al. [21,22] with the following modifications. For biofilm preparation single colonies were inoculated into 2 ml of LB medium and incubated for 16 h at 37°C while shaking at 250 rpm. Overnight cultures were diluted with salt-free LB medium (SF-LB) (1% Bacto tryptone, 0.5% Bacto yeast extract) to OD_660_ = 0.1. SF-LB containing 1.5% Bacto agar was sterilized by autoclaving at 120°C for 20 min, cooled and maintained at 60°C and 5 ml was poured into 36.7 mm Petri dishes. Dishes were left to solidify and dry with the lid closed for 30 min, and then dried with the lid open for another 30 min. A volume of 5 µl of the diluted overnight culture was then spotted onto the salt-free LB agar plate, and the plates were dried with the lid closed for 30 min. Plates were sealed with parafilm to prevent evaporation and incubated at 28°C in the LOTUS imaging system for up to 5 days. Antibiotics were added when required: 100 µg/ml ampicillin sodium, (FUJIFILM Wako Pure Chemical Corporation, Osaka, Japan) or 25 µg/ml kanamycin sulfate, (Nacalai Tesque, Inc., Kyoto, Japan) or both [19,20]. For sub-MIC experiments 8 µg/ml ampicillin was used with 1 µl spotting volume to approximate the conditions used by French et al. [14].

### LOTUS system design and assembly

#### 3D printing.

All 3D-printed components were designed from scratch or modified from public sources in OpenSCAD (version 2021.01) or FreeCAD (version 0.21.2, Windows 10, 64-bit) and printed using a Anycubic Kobra 2 Max 3D printer. G-code was generated using Anycubic Slicer (version 1.3.0). Actuator mechanism design files were downloaded from Thingiverse (https://www.thingiverse.com/thing:2707156). Components were printed using 1.75 mm black polylactic acid (PLA) filament (Creality, Shenzhen, China) with a 0.4 mm extruder, an extrusion temperature of 220°C, a bed temperature of 60°C and a lamination pitch of 0.2 mm. The total system cost was approximately $550 USD. Detailed breakdown of costs is shown in S1 Table in S2 File and STL files are provided in S3 File.

#### Image acquisition: Camera and optics.

For LOTUS images were captured on an Arducam (UCTRONICS-B0399) 64 MP camera module connected to a Raspberry Pi camera port via a 1 m ribbon cable (Fig 3J). For fluorescence imaging, we used a blue LED array (465 nm, LP-5B4SCHJ12, LED Paradise, Tokyo, Japan) with a 532 nm band-pass filter (FLH-532–10, Thorlabs, Newton, NJ, USA) for GFP detection and a green LED array (520 nm, LP-5SG4SCHJ15, LED Paradise) with a 665 nm long-pass filter (FGL665, Thorlabs) for mCherry detection. Images of growing biofilms were acquired at 20-min intervals over five days at a resolution of 2312 x 1736 pixel in JPEG format. The impact of image compression, which was minimal, is described in Supporting Information in S1 File (S12-S13 Figs). Complete camera parameters and settings are provided in S3 Table in S2 File.

**Fig 1:**
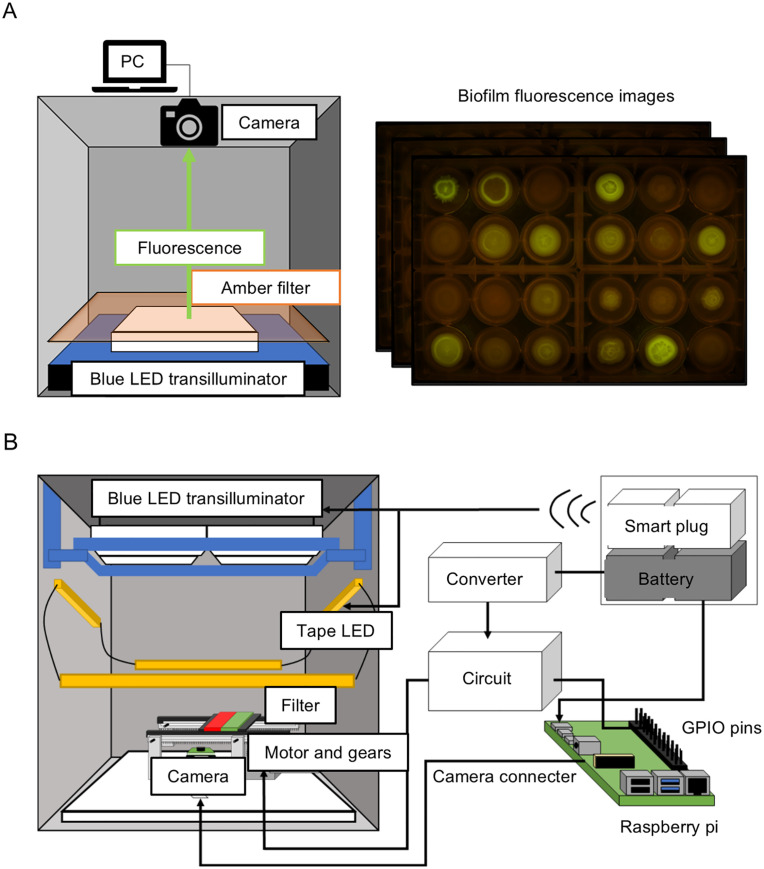
Single-mode imaging system prototypes. **(A)** In the first prototype, the shutter of the SLR camera is controlled with a computer and synchronized with a timer-controlled power-supply used for the blue LED transilluminator. Green fluorescence is selected via an amber filter. **(B)** In a more complex prototype variation, a Raspberry Pi computer is used to control a Pi camera, a rail-mounted optical filter change plate and blue Illumination module ON/OFF. Green and red fluorescence signals are selected via a band-pass filter (green) and a long pass filter (red). A smart-plug controlled white LED tape is used for bright-field epi-illumination.

**Fig 2:**
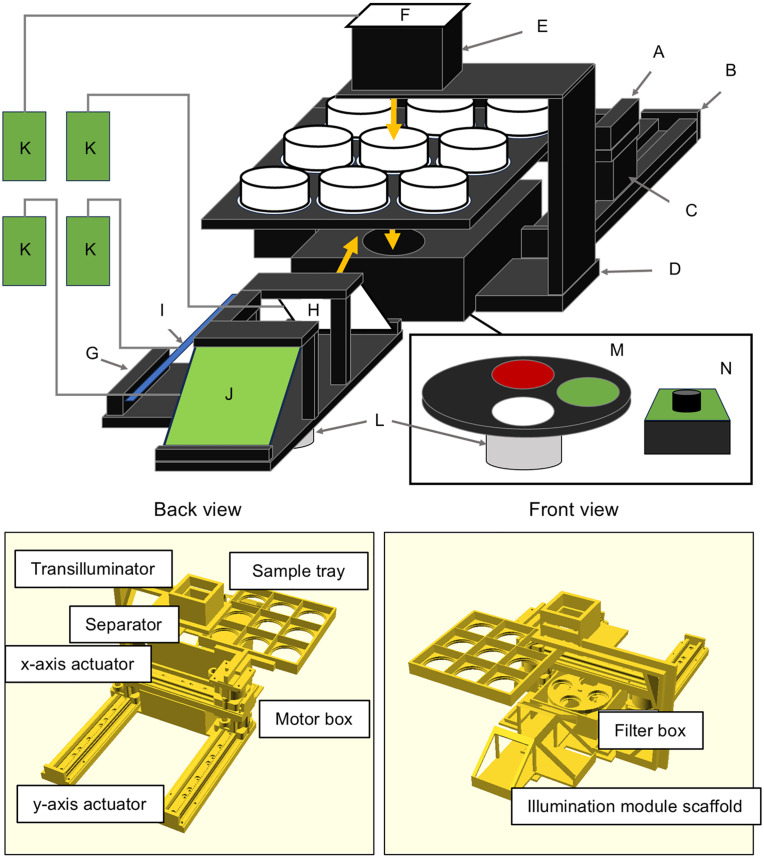
Overall design of LOTUS and its components. (A) x-axis actuator, (B) y-axis actuators, (C) stepping motor box, (D) supporting arm, (E) diffuser box, (F) white illumination module (for transillumination), (G) epi-illumination illumination module scaffold, (H) white illumination module (epi-illumination), (I) blue illumination module, (J) green illumination module, (K) relay modules wired to each illumination modules, (L) stepping motors attached to Illumination module scaffold and (M) filter wheel mounted with two optical glass filters and (N) camera module. The bottom panels reveal more details about 3D-printed components.

#### Sample positioning.

Sample positioning was achieved using stepping motors connected to linear actuators along the x and y axis. Actuator step size was measured using a Vernier micrometer (S1 Fig in S1 File, S1 Video). The number of steps was increased from 0 to 640 in increments of 32, and the travel distance after each increment was recorded. Linear regression was used to determine the relationship between motor steps and displacement distance, allowing calculation of steps required for a given displacement (S1 Fig in S1 File). In the current 9-well LOTUS configuration moving between the center of adjacent wells required approximately 617 steps on a newly built system. After further system use, the actual number of steps changed to 600 likely due to reduced friction from grease applied between the gears.

#### Comparative imaging.

Fluorescence detection sensitivity was compared for LOTUS, a Mithras LB940 multimode microplate reader (Berthold Technologies GmbH & Co. KG, Bad Wildbad, Germany) and a Amersham Typhoon laser scanner (Cytiva, Marlborough, MA, USA) using serial dilutions of fluorescein sodium salt (Nacalai Tesque, Inc., Kyoto, Japan) (10 ⁻ ³ to 10 ⁻ ⁹ M) dispensed into a 96-well plate in triplicates. Green fluorescence intensity was measured in each well on all instruments and values obtained without fluorescein (0 M) (background) were subtracted from all measurement values. To adapt LOTUS for use with microplates, a sample tray for the well plate was designed, printed, and attached to the 2D actuator (S6D Fig in S1 File). Then, all 96 wells were imaged in sets of 2 × 2 wells while varying the shutter speed. The fluorescence intensity per well was measured using the captured images. Measurement parameters are detailed in S3 File.

For imaging comparisons and validation, biofilms images were acquired using an Amersham Typhoon laser scanner (50 µm pixel length) under the same conditions as those used for imaging fluorescein using FluorescenceFMS and an Olympus SZX7 (DF PLAPO 1 × 4 objective) stereomicroscope equipped for transillumination (white LED board) and for fluorescence (485 nm green LED excitation and a band-pass filter (530–540 nm)). The camera (SWIFTCAM SC1603) was controlled using Swift imaging (ver 3.0) with a shutter speed of 10 ms and a gain of 500%.

### Quantitative image analysis

#### Biomass proxy.

Images were imported into Fiji/ImageJ. We estimated biofilm biomass using inverted pixel intensity (255 – measured intensity) of transillumination signals integrated over the whole biofilm area after background subtraction. We refer to this metric as “biomass proxy” throughout the manuscript, noting that while this measurement correlates with transmitted light attenuation through the biofilm and provides reliable relative comparisons under our experimental conditions, it should not be considered equivalent to spectrophotometric absorbance or optical density measurements. Due to extracellular matrix contribution, it can only provide an estimate of cell density. However, it provides a reliable biomass proxy for relative comparisons under similar experimental conditions and for samples in which no major quantitative changes in matrix compositions are expected. Under the conditions used here, the values were highly correlated with those obtained from fluorescence values of a constitutively expressed promoter (Fig 7). Additional information about the pros and cons of biomass quantification using various methods can be found in S3 File.

**Fig 3:**
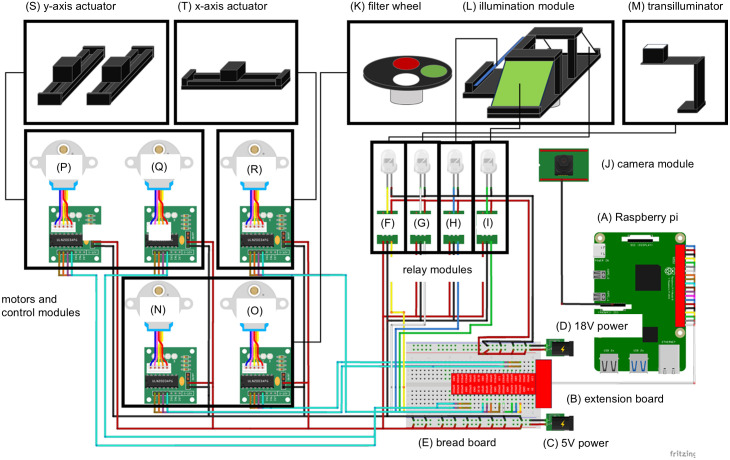
Overview of control and circuit components. **(A)** Raspberry Pi 4 model B, **(B)** GPIO extension board, **(C)** 5V DC power source for suppling power to stepping motor control module and relay module, **(D)** 18V DC power source for suppling power to illumination module, (E) bread board, (F-I) relay modules for different LEDs (Wayintop), (J) 64 MP camera (Arducam UCTRONICS-B0399), (K) filter wheel controlled by stepping motors, (L) illumination module controlled by stepping motors, (M) white LED transilluminator and support arm, (N-R) stepping motors (Shanxingan 28BYJ48) and control modules (ULN2003) attached to (S) y-axis actuator and (T) x-axis actuator or optical elements **(K-L)**. These schematics were generated using Fritzing software and MS PowerPoint.

**Fig 4:**
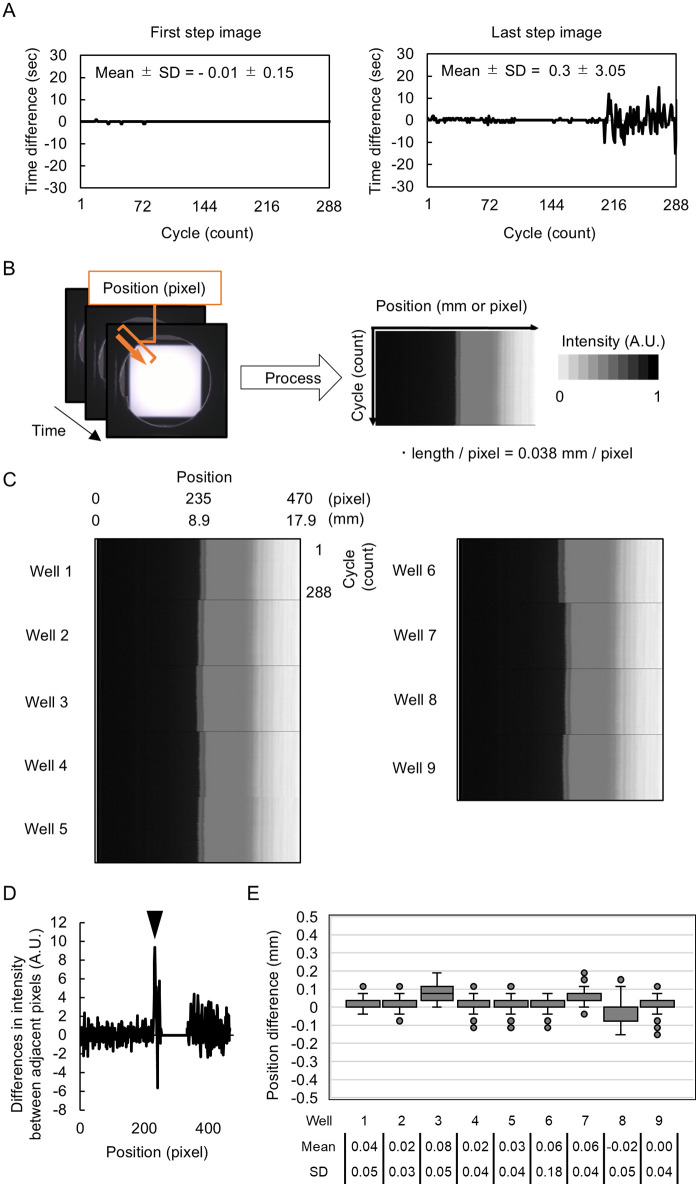
Benchmark tests for imaging time and sample position reproducibility (drift). **(A)** Variation in the image timestamps over 288 imaging cycles (count). The time stamp difference between transillumination images of well 1 (the first step of the imaging cycle) and white epi-illumination of well 9 (the last step of the cycle) is plotted as deviation from the expected cycle duration time (1200 sec) in seconds. **(B)** Analysis of well position alignment. Light intensity was measured along the orange arrow for each image, and kymographs (temporal heatmap) were generated to show edge position (mm or pixel) (horizontal axis), over time (288 x 12 min cycle, over 4 days) on the vertical axis. The pixel size was calculated from the diameter of the Petri dish. Pixel intensity is shown as normalized intensity. **(C)** Kymographs for each of nine well positions. **(D)** Detection of dish edge as maximum brightness variations position. **(E)** Well position variability. The dish edge position deviation from its initial position (t = 0) is shown for each well (n = 288 cycles). Box plots show the median, interquartile range (IQR) and whiskers extend to the most distant data point within 1.5 X IQR.

**Fig 5:**
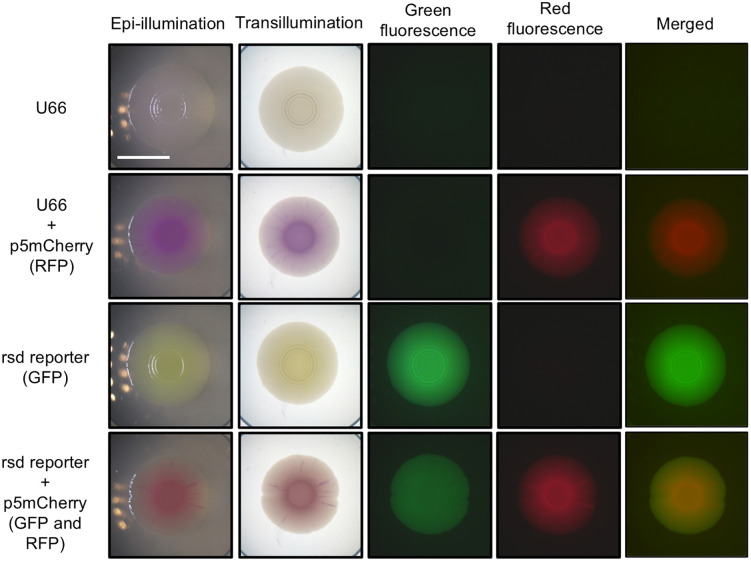
Multimode imaging of biofilms with LOTUS. Bright-field (epi-illumination and transillumination) and fluorescence images (green and red fluorescence channels) of biofilms containing one or two reporters were captured at biofilm growth experiment endpoint. Samples are: *E. coli MG1655* promoter strains U66 (promoterless), U66+p5mCherry, rsd, and rsd+p5mCherry.

**Fig 6:**
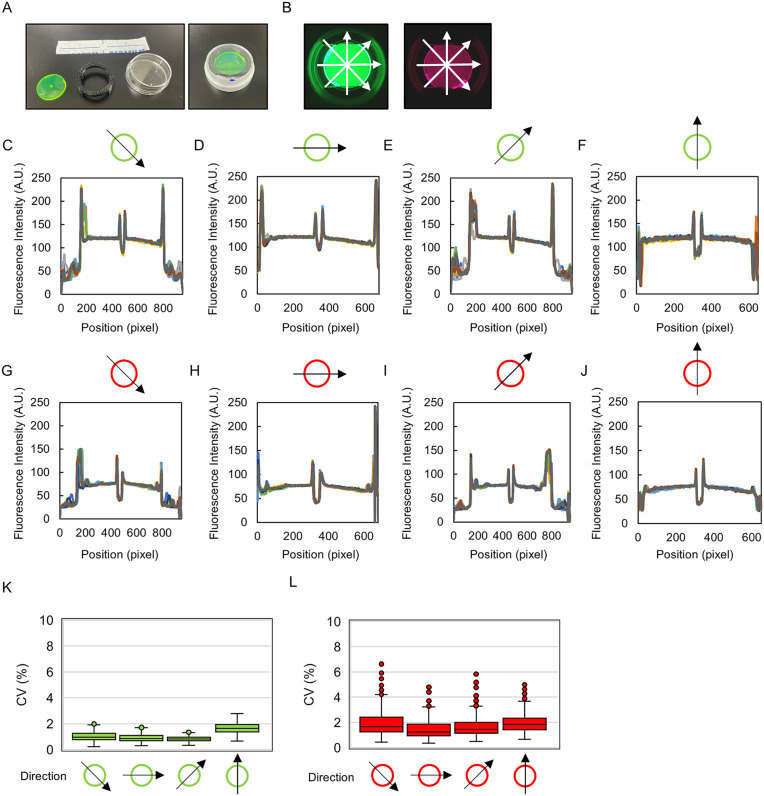
Evaluation of fluorescence intensity flatfield uniformity using alignment disks. **(A)** Alignment disk, 3D-printed adapter and 36.7 mm dish, shown separately and assembled. **(B)** Fluorescence intensity was measured at each well position using both green and red alignment disks at each pixel position along the four directions shown by arrows. **(C-J)** The resulting intensity profiles are shown for the green (C-F) and red (G-J) disks. **(K and L)**. Directional variation in CV values of pixel intensity over a 250-pixel window that excludes the central hole and disk edges for all well positions (n = 9). Box-and-whisker plots show the median, IQR, and whiskers extend to the most distant data point within 1.5 X IQR.

#### Background subtraction and normalization.

Extraction of inverted intensity and fluorescence intensity of biofilms from the acquired image dataset was based on the method of Takeuchi et al. [23]. The influence of green fluorescence was removed by extracting the red channel images using Split Channels. To correct for temporal changes or uneven transillumination, the Image Calculator tool of Fiji/ImageJ was used to subtract the initial image from all subsequent images. After applying Otsu thresholding to the subtracted images, the region of interest (ROI) signal (inner biofilm area) was binarized to derive the mean pixel intensity and area (signal). The mean pixel intensity value of the region outside the ROI was defined as background (or noise). For fluorescence images, the ROI obtained from the transillumination image was applied to the corresponding fluorescence image, and the average fluorescence intensities of the inner and outer regions of the biofilm were measured. The fluorescence intensity of the biofilm was determined by multiplying the difference between the mean fluorescence intensities of the inner and outer regions, by the area. Normalized fluorescence was calculated as the total signal intensity divided by the biomass proxy value at the corresponding time point.

#### Kymograph generation from biofilm images.

To visualize spatio-temporal biofilm dynamics in a single and condensed representation, we generated kymographs that show radial and temporal density profiles. Transillumination images were first converted to 8-bit grayscale and pixel intensity was inverted. Using the Line Selection tool, a line was drawn across the center of each biofilm to extract intensity values and generate the biofilm radial profile (Fig 5). For each image the resulting value arrays were chronologically arranged and imported into Excel. Each value in the array was background corrected by subtracting the initial image profile in each dataset. Kymographs were produced using the color scale function. For fluorescence images, the same process was applied to raw pixel intensity value of each image without intensity inversion.

### Generation of time‑lapse imaging videos

Time-lapse imaging videos of developing biofilms were generated from series of JPEG images using the AviUtl software. Video resolution was set at 2312 × 1736 pixel in full resolution with a frame rate of 60 fps using mp4 codec (S2 and S3 Videos). Additional methods are described in S3 File.

#### Focus drift and noise quantification.

A Laplacian filter was applied to image of the resolution target to emphasize edges. The processed image was used to obtain the profile of the ladder (Group 0, Element 1) using the Line Selection tool in Fiji, and the variance of all pixels was calculated.

To measure noise level in transillumination and fluorescence images, as for kymographs, images at each time point were normalized by subtracting the initial (t₀) image, and a line was drawn through the center of the biofilm. The standard deviation of the area outside the biofilm ROI was defined as noise. Then, for each time profile, regions with pixel intensity values ≥ 3X the noise level in transillumination images, were extracted and the mean value was used as the signal. Fluorescence noise values obtained from all nine wells were averaged to generate the graph. The signal-to-noise ratio (S/N) was calculated by dividing the measured signal value by the noise.

### Statistical analysis

All validations or experiments were performed in triplicates unless otherwise stated. Data are presented as mean ± standard deviation. Statistical significance was assessed using Welch’s t-test for comparisons between two independent groups and one-way ANOVA followed by Tukey’s test for comparisons among multiple groups using jamovi. Reproducibility of measurements was evaluated using the coefficient of variation (CV). The temporal CV was calculated from the mean and standard deviation of image profiles over time. The spatial CV was calculated from the mean and standard deviation of pixel intensities at each position over time, across different sample positions (wells). The limit of detection (LOD) was defined as five times the standard deviation of the mean blank value.

## Results

We are interested in exploring the development of bacterial biofilms and thus developed an automated system capable of simultaneous time-lapse imaging in bright-field and fluorescence modes to explore the dynamics of gene expression in growing biofilms using a collection of *E. coli* transcriptional reporter strains [19]. Each strain contains a different *E. coli* promoter driving the expression of GFPmut2. Our system evolved through various iterations. We originally imaged growing biofilms using a stereomicroscope, but the manual process made it difficult to obtain time-lapse information over extended periods of time and for multiple samples. Inspired by another project [15] a blue LED transilluminator (IORodeo, USA), an amber filter and a remotely controlled a digital SLR camera (Eos Kiss) were combined for time-lapse imaging of *E. coli* biofilms with different target promoters (Fig 1A). The simple prototype allowed to capture up to 24 biofilms growing in four separate 6-well plates. Results were qualitatively good but illumination uneven at the outer edge and wide-angle illumination resulted in image distortion and undesirable position-dependent reflections of fluorescent signals at the bottom of the plate. This system did not allow imaging in bright-field to observe morphology or quantify biomass. We then built a second prototype version by adding a filter exchanger (band-pass or long-pass) so that bright-field and fluorescence images could be taken automatically (Fig 1B). We used a Raspberry Pi computer to control the LED and a cheap Raspberry Pi camera. This version was suitable for observing biofilm structure and image in two fluorescence modes simultaneously. However, the wide field of view problem and the lack of a transillumination for biomass estimation were still limiting and we therefore decided to develop a third system, LOTUS that combined these capabilities.

### System overview

Instead of capturing multiple samples simultaneously, we opted to capture single, close-up images to avoid ghost signals and image distortion. This required additional mechanical components to position the samples over the camera. We therefore implemented a sample exchange mechanism using 3D-printed actuators to move the samples along the x-y dimensions. The completed fully automated imaging system integrates a nine-position sample tray, camera module white light transillumination for measuring biomass/cell density, white light epi-illumination for observation of biofilm surface structure, and blue and green LED/filter selection modules for epi-fluorescence imaging (Fig 2).

### System design

Most system components except for motors, LEDs, boards and filters are 3D-printed (Fig 2). The sample positioning mechanism is constructed using 3D-printed actuators with stepping motors to move the sample tray accurately over the camera (Fig 2A-2C). The optical components include two illumination modules and optical filters. For transillumination, a white LED source was attached to a supporting arm positioned immediately above the bottom of the sample plate to measure biomass (Fig 2D-2F). A rotating illumination module is positioned at a 35° angle and used for epi-illumination with a white LED array without filter (bright-field image) whereas blue or green LED arrays are used for fluorescence imaging (Fig 2G-2J). Emitted fluorescence is selected with a band-pass (green fluorescence) and a long-pass filter (red fluorescence) placed near the camera (Fig 2M-2N). A relay module is used to turn the light sources on and off (Fig 2K). Stepping motors control the positioning of the illumination module filter wheel (Fig 2L). A camera module connected to a Raspberry Pi is placed into an enclosure positioned below the transillumination white LED.

The sample tray is mounted on the motor box of the x-axis of a two-axis actuator. Horizontal movement in x and y directions is possible and controlled according to the number of steps of the stepping motor (Methods, S2A Fig in S1 File). The rotation angle of the stepping motor was estimated from the number of motor steps (516 steps per rotation, rotation angle at 90° = 128 steps). In addition, the illumination module scaffold and filter wheel are fixed to stepping motors connected in parallel, enabling synchronized rotation and positioning (S2B Fig in S1 File). The illumination module power is controlled via a relay module, and the camera is controlled from the Raspberry Pi. Details about all steps of the imaging cycle are provided in Methods.

The imaging cycle (bright-field, blue, and green fluorescence imaging) begins with the placement of the first sample well on top of the Raspberry Pi camera (S2 Table in S1 File). The white illumination module is switched on (Fig 3F), the image is captured (Fig 3J), and illumination module is switched off. The band-pass filter (GFP) is placed above the camera by rotating the filter wheel as the stepping motor turns (Fig 3K and 3N, S2B Fig in S1 File). Similarly, a stepping motor rotates the illumination module scaffold to position the blue LED array toward the sample (Fig 3L and 3O), it is switched on (Fig 3H) and image captured before the module is switched off. Next, the same process is repeated for the long-pass filter (RFP) and green LED array, followed by the empty filter slot and white LED array for transillumination. Finally, the x- and y-axis actuators are moved to position the next well above the camera (Fig 3P-3R). This sequence is repeated for all nine well positions. With the current system settings, one cycle requires about 12 min (S2 Table in S2 File).

In the coming sections we describe how we first validated technical performance, then demonstrated biological applications.

### System validation and benchmarking

#### Timing and positioning accuracy.

Our system was designed to monitor biofilm development and gene expression by imaging the same samples repeatedly over several days. Under such conditions, proper synchronization and reproducibility of image capture time and well positioning at each cycle of imaging is important for reliable analysis.

To verify timing accuracy, the system was operated with an empty dish loaded in each of the nine well positions and then imaged every 20 min for a total of four days. The time stamps of the first transillumination image of Well 1 and the white epi-illumination image of Well 9 were extracted and the time difference between the actual time stamp and the expected time stamp was calculated as follows and plotted (Fig 4A).

Time difference (sec) = measured interval from time stamps (sec) – 1200 sec

Timestamp data from Well 1 were highly consistent with the expected values (S2 Table in S2 File) across 288 cycles, with mean deviation of 0.01 ± 0.15 sec. The time stamp for Well 9 showed slightly more pronounced drift with mean deviation of 0.3 ± 3.05 sec and maximum of 15 sec after 3 days (Fig 4A). However, this drift remains negligible (< 2% deviation) considering the total cycle time of 1200 sec.

To evaluate positional accuracy, bright-field images from all wells were analyzed by measuring pixel intensity along an arrow to create kymographs and visualize position shifts over time (Fig 4B, 4C). The distance in pixels from the arrow’s origin to the position where pixel intensity changed abruptly (dish edge) was measured for all images and compared to the initial position (Fig 4D-4E).

Kymographs generated from the images taken in each well showed minimal drift over 288 cycles (4 days) (Fig 4C). The maximum positional deviation was approximately 7 pixels (or 0.3 mm) across all wells (Fig 4E). Together, these results show that accurate time capture and sample positioning is possible and reliable over several days of continuous operation.

#### Resolution evaluation.

To verify the optical resolution of the camera, a test target (R1L1S7P, Thorlabs) was mounted on a scaffold placed in a Petri dish and imaged with white light transillumination (S3 Fig in S1 File). From captured images, we generated intensity profiles across line pairs for each element (small number) within each group (large number) (S4 Fig in S1 File) and the resolution was calculated using the target specifications from Thorlabs. https://www.thorlabs.com/newgrouppage9.cfm?objectgroup_id=4338&pn=R1L1S7P Resolution cutoff was set at the highest spatial frequency for which the Michelson contrast remained ≥10%, a commonly used cut-off value for stripe discriminability. This condition matched Group 2, element 6, which corresponds to 7.13 lines/mm, or a line pair spacing (resolution) of 140 µm. Resolution was therefore estimated as < 0.14 mm.

#### Quality control for LEDs and optical filters.

After validating the technical optical performance of LOTUS, we next evaluated its utility for biological applications by monitoring biofilm development with transcriptional reporter strains expressing fluorescence proteins [19]. For this, we used a promoterless negative control strain (U66) which does not express GFPmut2, and the *rsd* promoter strain previously shown to constitutively express high levels of GFPmut2 [14]**.** To generate dual-reporter strains, the U66 strain and rsd promoter strains were transformed with the p5mCherry plasmid which constitutively expresses mCherry [20]. The strains were cultured on salt-free LB agar medium for 5 days and biofilms were imaged in four modes (Fig 5) revealing structure (epi-illumination), biomass (transillumination) and clearly distinguished patterns of fluorescent protein expression. Fluorescence intensity was also quantified at each pixel position along arrows transecting the biofilm center to quantify signals in space (S5 Fig in S1 File).

This analysis revealed minor crosstalk in which some GFP signal was detected in the red channel of rsd biofilms (S5B-S5C Fig in S1 File). As shown in S21 Fig in S1 File, GFP emission overlaps with the 665 nm long-pass filter window and may explain this phenomenon. Both fluorescent proteins were detected in the rsD + p5mCherry biofilms although GFP signal intensity was reduced compared to the rsd single-reporter strain possibly due to metabolic burden of dual-plasmid maintenance or optical cross-excitation effects. Together, these results demonstrate that the selected LED/filter combinations are appropriate for dual-fluorescence detection with commonly used fluorescent proteins, though users should remain aware of minor cross-talk effects.

#### Illumination uniformity.

LOTUS is operated by sequentially positioning sample wells on top of the camera through movement of the sample tray, and deviation in the well positioning can lead to differences in the imaging conditions. We therefore checked whether the detected fluorescence signals could be compared across wells. Alignment disks emitting green and red fluorescence (ADF-7 and ADF-10, Thorlabs) were placed in a Petri dish together with a scaffold made by a 3D printer and the lid immobilized with Parafilm (Fig 6A). The dish was placed in the sample tray, positioned above the camera, and both green and red fluorescence images were captured for all nine sample positions. The light intensity of each pixel was analyzed along the four directions (Fig 6B).

The fluorescence intensity patterns detected in all wells overlapped for all image analysis directions with large peaks corresponding with the disk edges and central hole (Fig 6C-6J). In addition, spatial variation in fluorescence signal across the disk center (± 250 pixel region away from the central hole) was low (median CV < 2%, n = 9) in all directions (Fig 6K-6L). These results show that illumination is relatively uniform across well position and sample area.

#### Comparison with commercial instruments.

To evaluate the potential of this system, we compared its sensitivity and reproducibility with commercially available instruments, a standard fluorescence plate reader and laser scanner.

#### Sensitivity analysis using fluorescein.

To compare sensitivity, we used different known concentrations of fluorescein and analyzed their signals on LOTUS, a Mithras LB940 plate reader and an Amersham Typhoon laser scanner. The LOD was 1.20 × 10 ⁻ ⁸ M for LOTUS modified with a microplate tray, 1.17 × 10 ⁻ ⁸ M for the plate reader, and 3.98 × 10 ⁻ ¹⁰ M for the laser scanner (S6 Fig in S1 File). LOTUS LOD for fluorescein is comparable to the plate reader but about 30-fold less sensitive than the laser scanner.

#### Uniformity and reproducibility within biofilms.

Images of rsd promoter strain biofilms cultured for three days were acquired using LOTUS and laser scanner under transillumination or OD mode (Typhoon) and green fluorescence mode (S7A-S7B Fig in S1 File). To assess radial uniformity, intensity profiles were extracted using Line Selection tool in Fiji through the center of the biofilm from four directions and aligned at the position where the inverted intensity or fluorescence intensity began to increase (S7C Fig in S1 File). The aligned profiles were plotted relative to the horizontal analysis direction (S8 Fig in S1 File). Additionally, the spatial CV per pixel was calculated for each direction for all pixels (S7D-S7E Fig in S1 File).

In all analyzed directions, the intensity from the center to the edge of the biofilm was symmetrical and similar for both instruments (S8 Fig in S1 File). These results indicate that the inverted intensity and fluorescence intensity distribution of the biofilm showed little difference across analytical directions. Overall, biofilm intensity profiles from LOTUS showed directional uniformity (median CV < 2%, S7 Fig in S1 File) comparable to a commercial laser scanner.

#### Evaluation of reproducibility during imaging.

Next, we evaluated whether imaging results using LOTUS were reproducible after continuous operation for 3 days. While minor focus drift cannot be completely excluded (S3 File), we did not observe major fluctuations in Laplacian variance during time-lapse image analysis, while we did after a manually triggered focus change (S9B-S9C Fig in S1 File). In transillumination mode, the area and mean intensity of the ROI within a PLA disk remained constant during time-lapse imaging (area: 99.97 ± 0.25%, mean intensity: 99.62 ± 0.16%, t0 = 100%) (S10 Fig in S1 File). Similarly, the average relative fluorescence intensity within the green alignment disk (99.71 ± 0.09%, t0 = 100%) and the intensity profiles in four directions also remained constant (median CV < 1%) over the 3-day period (S11 Fig in S1 File). Overall, focus stability, area measurements, and fluorescence intensity remained stable over three days of continuous operation. More details in S3 File.

### Condensation and temperature fluctuations

During prolonged time-lapse observation in Petri dishes, water evaporation from the agar surface can lead to condensation on the lid surface and image blurring [10]. However, during normal LOTUS operation no obvious condensation was observed except after stopping the system. This suggests that condensation is suppressed during operation due to slight continuous heating of the Petri dish lid (S14 Fig in S1 File) by illumination. However, as seen in S15 Fig in S1 File, LOTUS operation did not affect the temperature inside the incubator. Further details regarding condensation can be found in S3 File.

### Biological demonstration of LOTUS performance

#### Multimode time-lapse biofilm imaging.

To demonstrate LOTUS ability to capture the growth and dynamics of fluorescent protein expression in living biofilms, bright-field epi- and transillumination, green and red fluorescence imaging were tested with dual-plasmid reporter strain (rsd + p5mCherry). The time-lapse imaging videos of nine technical replicates revealed no obvious changes between wells over five days, showing good reproducibility (S2 Video).

**Fig 7:**
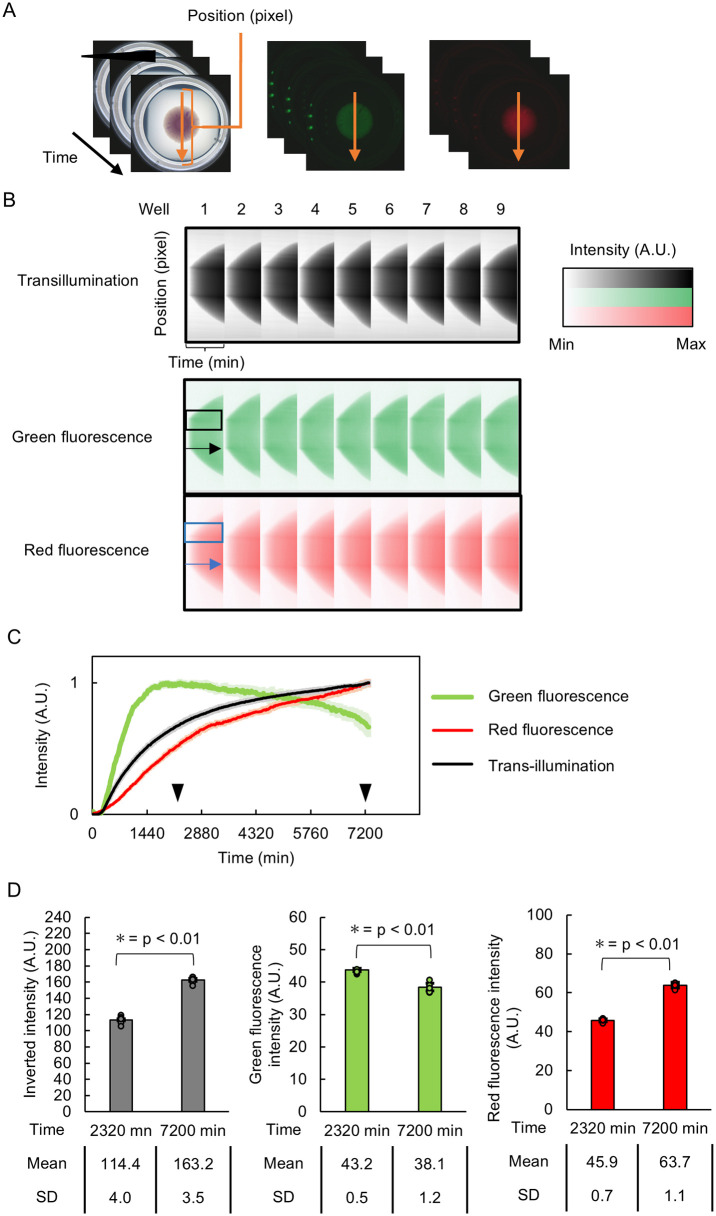
Spatio-temporal fluctuations of fluorescent reporters in growing biofilms. **(A)** Time-lapse images collected during the growth of *E. coli* biofilms expressing GFPmut2 and mCherry (Fig 5) were analyzed along the orange arrow and the pixel intensity was measured. **(B)** Kymographs showing pixel intensity along the spatial (horizontal axis) and time (vertical axis) dimensions. Color intensity is displayed as the relative intensity in either bright-field (transillumination), green (GFPmut2) or red (mCherry fluorescence). **(C)** Transmitted light or fluorescence intensity of *E. coli* biofilm growth. Shading represents the SD. **(D)** Integrated intensity values for transillumination or fluorescence signals are plotted for the two time points highlighted by arrowheads in **(C)** (2320 and 7200 min). The error bars show the mean ±SD (n = 9, technical replicates).

Spatio-temporal analysis (kymographs) revealed the dynamics of fluorescent protein distributions (Fig 7A-7B). GFP fluorescence intensity initially increased throughout the center of the biofilm, expanding radially before decreasing in the central region after 2320 min (Fig 7C). On the other hand, mCherry fluorescence increased continuously without subsequent decrease. Quantification showed that biomass proxy (transillumination) and mCherry fluorescence increased significantly between 2320 and 7200 min (peak and endpoint, respectively) while GFP fluorescence decreased (p < 0.01, Welch’s t-test, n = 9, Fig 7D).

The apparent GFP fluorescence decrease in dual-reporter analysis may reflect actual regulation, but technical artifacts cannot be excluded. As mentioned earlier, minor spectral cross-talk was detected despite the use of a 665 nm long pass filter (S21 Fig in S1 File). Further validation would be needed to clarify this but overall, LOTUS demonstrated reproducible quantitative detection of temporal changes for both fluorescent reporters.

#### S/N ratio measurement.

Noise levels and S/N ratio in fluorescence images from Fig 7 were calculated from the mean intensity within the biofilm area (ROI) and the standard deviation of the intensity outside the biofilm (S16 and S17 Figs in S1 File). The noise level was 1.0 or less at all measurement points in the green and red fluorescence images (S16 Fig in S1 File). S/N exceeded 3 at 380 min in all replicates with noise levels remaining stable (green fluorescence mode CV = 8.03%, red fluorescence mode CV = 8.52%) throughout 5 days of imaging (S17 Fig in S1 File). These results show stable and low noise levels during long-term imaging using LOTUS. Signal detection before 150 min was limited by near-background fluorescence levels and below the threshold sensitivity of the image analysis process using Fiji. This could potentially be improved by adjusting LED power, camera settings or by optimizing image analysis workflow for weak signals.

#### Monitoring sub-MIC ampicillin response.

**Fig 8:**
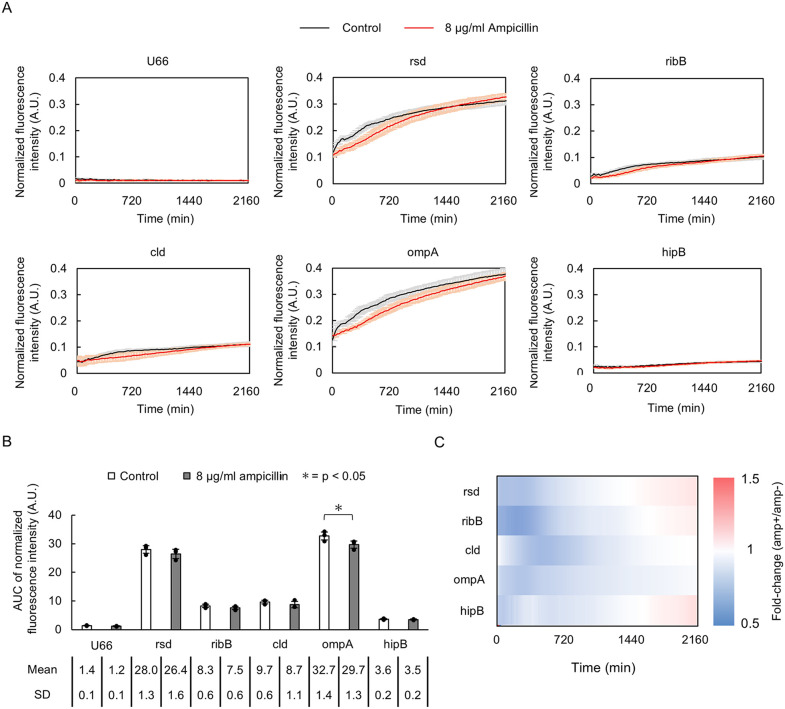
Effect of sub-MIC ampicillin treatment on GFP expression level, for selected promotor strains in growing *E. coli* biofilms. **(A)** Temporal changes in biomass proxy normalized fluorescence intensity for (U66 promoterless) and five selected promoter strains. Error bars indicate the standard deviation (n = 3, biological replicates). **(B)** Change in integrated fluorescence intensity over the whole biofilm area. The AUC was calculated from the first time point at which normalized fluorescence intensity was detected until experimental endpoint (n = 3). The bar shows the mean and error bars indicate SD (n = 3). **(C)** Heatmap of fold-change in normalized fluorescence intensity during biofilm development (ampicillin presence vs absence).

To evaluate whether LOTUS can quantitatively measure GFP expression level, we investigated the effect of sub-MIC ampicillin treatment on biofilms. The six promoter strains tested were: U66 (promoterless control), *rsd* (regulator of sigma D, strong constitutive), *ribB* (riboflavin biosynthesis), *cld* (chlorite dismutase), *ompA* (outer membrane protein A), and *hipB* (antitoxin/DNA-binding transcriptional repressor, persister formation). Biofilms for each strain were grown on salt-free LB agar medium with or without addition of a sub-MIC concentration of 8 µg/ml, for three days at 28°C. To account for delayed growth in the sub-MIC group, growth curves were temporally aligned by setting t = 0 as the onset of detectable growth (defined as the earliest time point where binarized biofilm area began stable increase following its minimum value). Results showed that normalized fluorescence intensity (intensity/biomass proxy value) increased over time in all promoter strains except the promoterless U66 strain (Fig 8A). U66 and other promoter strains showed trends toward reduced AUC with ampicillin treatment; however, except for *ompA* (p < 0.05, n = 3, Welch’s t-test, Fig 8B), these differences were not significant, likely due to limited statistical power. Temporal analysis of fluorescence intensity (fold-change with ampicillin vs without) showed an initial decrease (0–720 min) followed by an increase in signal intensity at later time points (T > 1500 min) for most strains (Fig 8C). This biphasic response might imply adaptative responses to cell wall stress.

We then compared and quantified end-point biofilm transillumination and green fluorescence total intensity values between LOTUS and a stereomicroscope (S18 Fig in S1 File). Fluorescence measurements for LOTUS correlated with those of the stereomicroscope, confirming reliability of quantification. On the stereomicroscope, all promoter strains showed fluorescence intensities that differed significantly from U66 (n = 3, p < 0.01, one-way ANOVA followed by Tukey’s test). On LOTUS, all strains except *hipB* showed similar significant differences from U66. hipB fluorescence intensity was not significantly different from U66 (p > 0.01, S18B Fig in S1 File). While under the conditions used, the microscope showed higher S/N for the fluorescence intensity, the differences between strains were consistent on both systems.

#### Evaluation of phototoxicity during time-lapse imaging.

Time-lapse imaging can result in phototoxicity [24]. Therefore, we evaluated the phototoxicity during time-lapse imaging with LOTUS on *E. coli* biofilms using an illumination scheme shown in S19A Fig in S1 File. Briefly, biofilms were imaged once a day, following: 1) no illumination (control, imaged only at 24-h intervals), 2) white LED exposure only, 3) white + blue LED exposure, and 4) white + green + blue LED exposure at each imaging cycle (S19A Fig in S1 File). The results showed trends toward decreased biofilm area, biomass proxy, and fluorescence intensity with increased illumination exposure (S19B-S19C Fig in S1 File). However, these trends were not statistically significant at 5 days (n = 3, p > 0.05, one-way ANOVA) and there was no significant difference in the final viable cell count (S19D-S19E, S20 Figs in S1 File). Therefore, while phototoxicity during prolonged imaging using LOTUS could not be ruled out, it was not significant under the conditions used. Additional details are provided in S3 File.

## Discussion

We developed and validated LOTUS, a multimode automated time-lapse imaging system, for measuring growth, morphological changes, and gene expression dynamics in bacterial biofilms. LOTUS improves upon our earlier laboratory imaging prototypes by implementing epi-illumination to reduce reflection artefacts and modular LED arrays and filters for flexible wavelength selection. The presented version of LOTUS captures up to nine biofilms over time in bright-field and dual-fluorescence mode offering reasonable throughput. The system is built from commercially available single-board computers, inexpensive electrical modules, and 3D-printed components considerably reducing costs (>90%) compared to commercial alternatives. Good temporal reproducibility was maintained over a 5-day measurement period with little focus drift (S9-S11 Figs in S1 File) and biological replicates showed consistent fluorescence dynamics for different promoter activities dynamics (Fig 8). End-point fluorescence signals were also consistent with a laser scanner and stereomicroscope (S7 and S18 Figs in S1 File).

### Limitations of LOTUS

Well positioning accuracy was good (±0.3 mm) and sufficient for macro-scale biofilm imaging but may require corrections for higher magnification applications or smaller samples. When this is of concern, such deviations can be reduced using alignment algorithms during image analysis. Both positioning drift and imaging cycle time (12 min for 9 wells) could be reduced by using higher-voltage and higher-torque stepper motors and commercially available actuators.

The measured resolution of LOTUS (< 0.14 mm) is limited but appropriate for the intended macro-scale applications (1–25 mm). Higher magnification is possible using alternative cameras (e.g., Raspberry Pi HQ camera and lens) and appropriate lens adapters. The use of higher-specification single-board computers could enable faster and higher-resolution imaging with real-time image analysis for automated sample alignment corrections.

Background fluorescence signals increase in the biofilm periphery appearing as shoulders outside the biofilm edge (S5 Fig in S1 File). These shoulders originate from reflected signals and increase as biofilms grow necessitating time-point specific background subtraction from adjacent regions. This phenomenon may introduce minor systematic uncertainty at later times points and for stronger fluorescent signals but should not affect relative comparisons within experiments.

The observed slight radial fluorescence intensity gradients visible in the raw intensity profiles (Fig 6C-6J) are common for single-lens cameras and can be corrected computationally if necessary for accurate quantitative applications.

In this study, images were collected in JPEG format due to current system constraints. Based on our analysis, JPEG compression introduces quantitative errors of approximately 5% (S12-13 Figs in S1 File, S3 File), which is acceptable for semi-quantitative and comparative analysis but may affect spatial pixel-level measurements. Uncompressed formats are recommended for precise quantitative analysis.

### Comparison of performance with commercially available hardware

We found that the sensitivity of LOTUS in fluorescence mode was comparable to a commercial plate reader but about 30-fold lower than a laser scanner likely due to differences in detectors (CMOS vs PMT) and illumination (LED vs laser). This LOTUS version is adequate for moderate to strong GFP expression monitoring (S3 File).

### Comparison with other open-source laboratory solutions

Several other open-source imaging systems have been developed for time-lapse imaging [5,10,14–18]. While they may excel at specific tasks, they do not integrate the multi-sample, multi-mode imaging features of LOTUS for comprehensive biofilm analysis. Some such as Picroscope [17] allow multi-sample imaging but in a single bright-field illumination mode. Others enable fluorescence but lack bright-field or automated filter exchange [14–15] or can only image single samples [5]. Finally, while openIVIS combines multi-mode imaging, it uses RGB LEDs and lacks customizable filter selection [16,18].

### Cost and maintenance

While detailed comparative cost-effectiveness analysis is not presented, LOTUS development costs (<$600 USD, S1 Table in S2 File) represent greater than 90% reduction compared to a basic commercial system with similar functionality (e.g., Celloger Nano, clear-field + green fluorescence mode only, without well alignment functionality, $8850 USD). Replacement costs for most components are minimal (<$200 USD) and no obvious degradation was observed over six months of continuous operation except for gradual LED light intensity decrease which does not affect relative measurements.

### Spatio-temporal fluctuations in fluorescence intensity in growing biofilms

LOTUS successfully captured spatio-temporal patterns of fluorescent protein expression within growing biofilms over 5-day periods. However, we observed reduced GFP intensity in the dual-reporter strain (rsd + p5mCherry) compared to the single-reporter strain (rsd), particularly near the center of the biofilm (Fig 7D). This persisted even when illumination was minimized (S5 Fig in S1 File) ruling out photobleaching as the primary cause. One possible explanation is that the plasmid copy number differences (low copy pUA66 (GFP) reporter vs high-copy p5mCherry (RFP)) may result in preferential recruitment of transcriptional machinery components (RNA polymerase and sigma factors) to mainly high-copy plasmids [19,20]. Alternately, spectral overlap between mCherry excitation and GFP emission may result in energy transfer processes in which GFP-emitted photos excite mCherry instead of reaching the camera (S21 Fig in S1 File) [25]. Such findings emphasize the need for careful controls and consideration of possible spectral interference when interpreting dual-reporter data especially in dense bacterial populations like biofilms.

Additionally, discrepancies between biomass proxy (transillumination) and mCherry fluorescence intensity (Fig 7C) likely reflect extracellular matrix contributions to biomass measurement by optical density [26] or visible light absorption by mCherry resulting in overestimated biomass. To address these issues, we are developing longer-wavelength illumination detection methods.

### GFP expression level within growing biofilms

LOTUS reliably detected temporal changes in GFP expression levels within biofilms exposed to sub-MIC ampicillin, with good reproducibility across biological replicates (Fig 8). Sub-MIC ampicillin treatment initially reduced fluorescence intensity across all promoter strains (0–720 min), followed by a recovery near control levels or higher after 2160 min. Since the constitutive rsd promoter displayed similar dynamics, fluctuations may reflect cell number changes rather than actual promoter activity responses [14].

Although the promoter strains were selected to represent a diversity of responses to ampicillin based on previous analysis using the same strains [14], our results quantitatively differ likely due to differences in growth conditions affecting ampicillin stress response [27,28] rather than contradicting previous findings. These include our use of biofilm-inducing conditions (salt-free LB at 28°C) instead of LB at 37°C, large biofilms size vs small colonies, and our longer experiments that ran for 36 hours vs 18 hours.

The reproducibility of our results demonstrates the capacity of LOTUS for comparative quantitative analysis of biofilm gene expression dynamics while highlighting their dependence on environmental perturbations. In addition, our observations show that fluorescence changes reflect the interplay between biomass, cell density and reporter expression and LOTUS simultaneous multi-mode imaging can enable deconvolution of these factors and better characterization than single-mode systems.

### Effect of LED illumination

Phototoxicity assessment revealed modest but non-significant decreases in biofilm area, biomass and fluorescence upon prolonged exposure but no effect on viable cell counts (S19-S20 Figs in S1 File). Blue light can inhibit biofilm formation, and the observed decrease in biomass might be linked to reduced extracellular matrix production which may be relevant for extended time-lapse experiments [24]. These effects may be negligible for relative comparisons under standardized illumination protocols but users should optimize imaging intervals to minimize cumulative light exposure for sensitive application.

LED operation reduced condensation accumulation on the lid of the Petri dish (S14 Fig in S1 File), improving image quality without requiring lid removal or humidity control. As dishes are sealed with parafilm, agar plate drying conditions prior to sealing may influence condensation. Incubator temperature was stable during the whole imaging cycle suggesting minimal thermal impact from the system.

### Customizability

LOTUS modular design can allow straightforward adaptation to different sample formats such as 96-well plates by modifying the sample tray and adjustment well positions. However, light containment may become an issue with narrow wells to prevent cross-well illumination and phototoxicity, as is commonly implemented in plate readers. More details in S3 File.

## Conclusion

LOTUS combines 1) automated multi-sample imaging (9 wells), 2) customizable multiwavelength illumination with automated filter exchange and 3) simultaneous bright-field and dual-fluorescence modes. This combination of features, previously unavailable in similar open-source systems, enables comprehensive macro-scale phenotypic characterization of biofilms (e.g., morphology, density, and gene expression). However, it trades higher resolution performance for low cost and versatility and as such is complementary to high-end microscopy-based systems when higher-resolution or finer optical performance is required.

The capabilities of LOTUS should be applicable to diverse biological applications beyond biofilm imaging, including other types of microbial colonies or communities, plant tissue cultures, etc. with limited modifications. Future feature development could include adaptative imaging by real-time image data analysis or built-in environmental control.

In conclusion, LOTUS provides a reliable, reproducible and customizable platform for multi-mode and macro-scale time-lapse imaging at a fraction of the cost of commercial systems. As such it addresses a gap in accessible instrumentation for laboratory with limited resources and encourages efficient allocation of research funds. Its open-source design invites further community adaptation and improvements that will continue to improve access to quantitative biology tools.

5mCherry, rsd, and rsd + p5mCherry strain. Green fluorescence images were obtained using a green band-pass filter and blue LED. Red fluorescence images were obtained using a long-pass filter and green LED. Merged images were created by overlaying green and red channel fluorescence images. LED and filter spectral properties are shown in S21 Fig in S1 File. The scale bar indicates 1 cm.

## Supporting information

S1 File Supporting Figures Sakai et al. (PDF)

S2 File Supporting Tables Sakai et al. (PDF)

S3 File Supporting information Sakai et al. (PDF)

S1 Video Measurement of the distance traveled by the actuator as a function of the number of steps. (MP4)

S2 Video Time-lapse video of growing *E. coli* biofilms expressing GFPmut2 and mCherry. Growth of nine biofilms captured in four illumination modes, corresponding with images used in Fig 7. (MP4)

S3 Video Time-lapse video of *E. coli* biofilms growing under various illumination conditions. Twelve biofilms corresponding with images used in S19 Fig. (MP4)
