# Supplementary material for: LOTUS: A low-cost time-lapse automated imaging system for spatio-temporal analysis of microbial colony or biofilm development
Source: PLoS One. 2026 Jan 23;21(1):e0339652. doi: 10.1371/journal.pone.0339652 (PMC12829848; doi:10.1371/journal.pone.0339652)
Supplement: S1 File — (PDF) [file pone.0339652.s001.pdf]

Supporting Figures

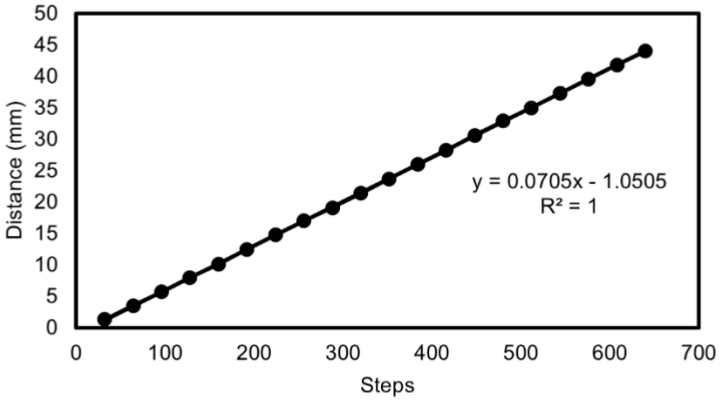

S1 Fig. Measurement of motor steps and travel distance. The number of steps travelled by the motor was successively increased by 32 and the linear distance traveled measured (S1 Video). The resulting linear regression equation was used to calculate the number of steps required to move between sample positions on the sample tray.

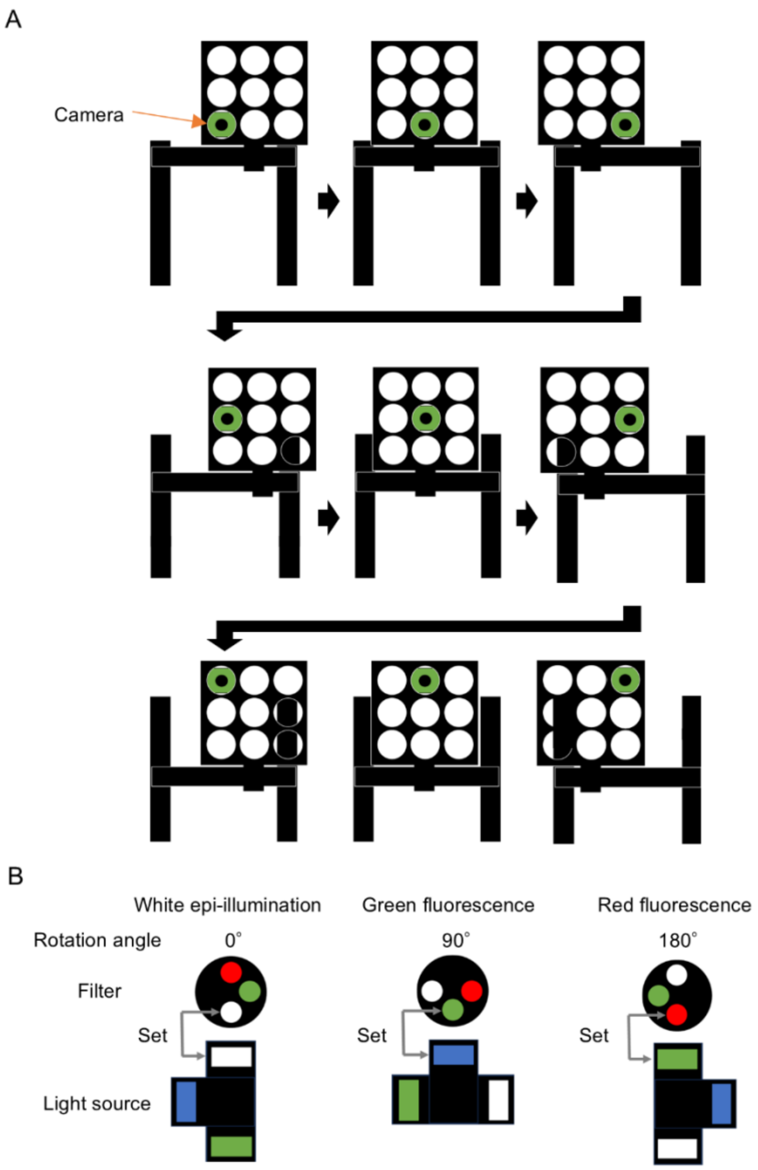

S2 Fig. Diagram of system component movement and operation. (A) Top view of wells. The position of the sample tray is controlled by x-axis and y-axis actuators, allowing all nine wells to be positioned directly above the camera. (B) Top view of filter disk and light source rack. The filter wheel and illumination module scaffold rotate in tandem. For white light epi-illumination, no filter is used. For green fluorescence imaging, a blue LED and a green band-pass filter are used. For red fluorescence imaging, a green LED and a red long-pass filter are used. Filters are positioned in front of the camera.

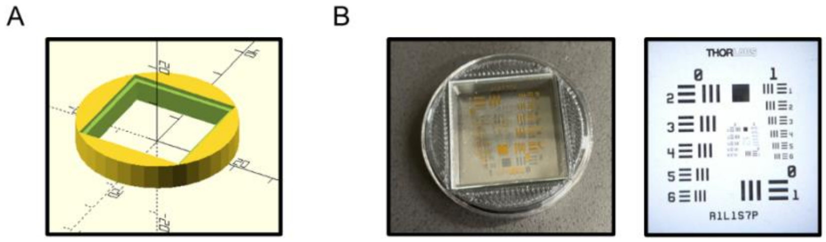

S3 Fig. Preparation of target for resolution test. (A) A scaffold was designed and 3D-printed to hold a (B) Thorlabs resolution test target (R1L1S7P) placed inside of Petri dish. The target was imaged in transillumination mode.

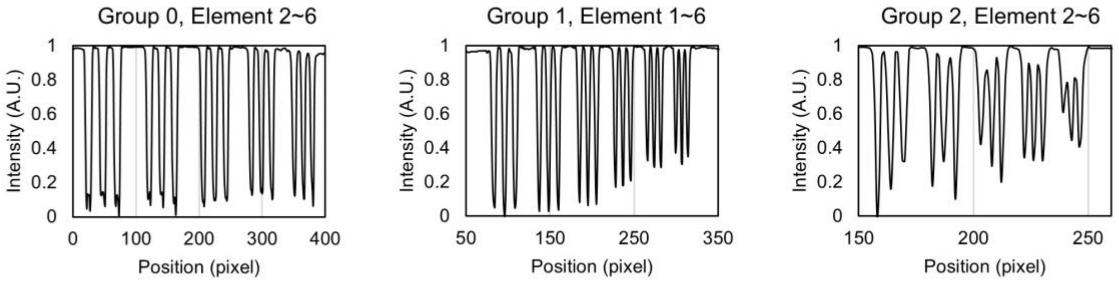

S4 Fig. Resolution tests. Light intensity profiles of each resolution bar group imaged by transillumination. The light intensity at each pixel (arbitrary units) was measured along a line crossing line triplets in different resolution group. Group 2, element 6 shows a Michelson contrast >10%, equivalent to a resolution of < 0.14 mm (Methods).

S5

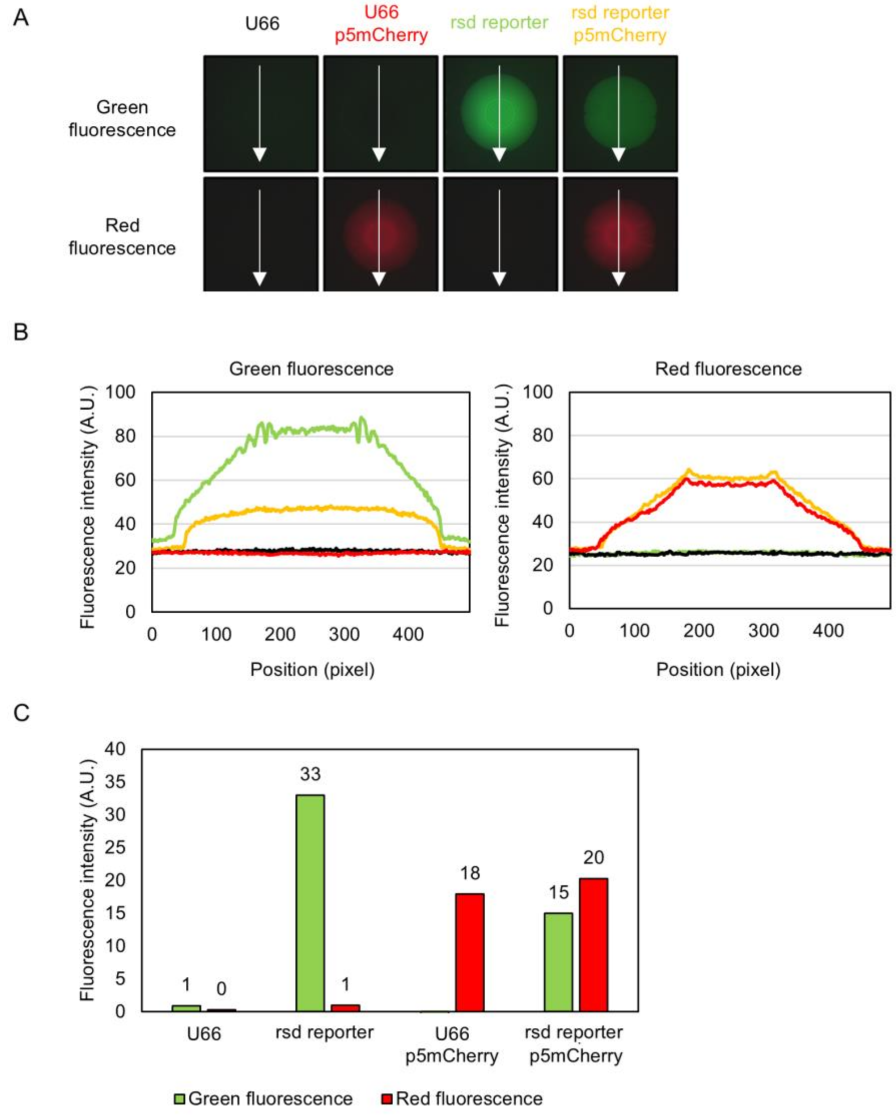

S5 Fig. Fluorescence signals for different promoter strains. (A) Biofilms were grown in the dark (no imaging) and a single image was taken at 5 days and analyzed. Fluorescence intensity was measured along the arrow through the center of the biofilm and (B) quantified for the indicated imaging modes. Colored lines represent the U66 (black), rsd (green), U66+p5mCherry (red), and rsd+p5mCherry (orange) strains. (C) Integrated biofilm fluorescence intensity for the indicated strains.

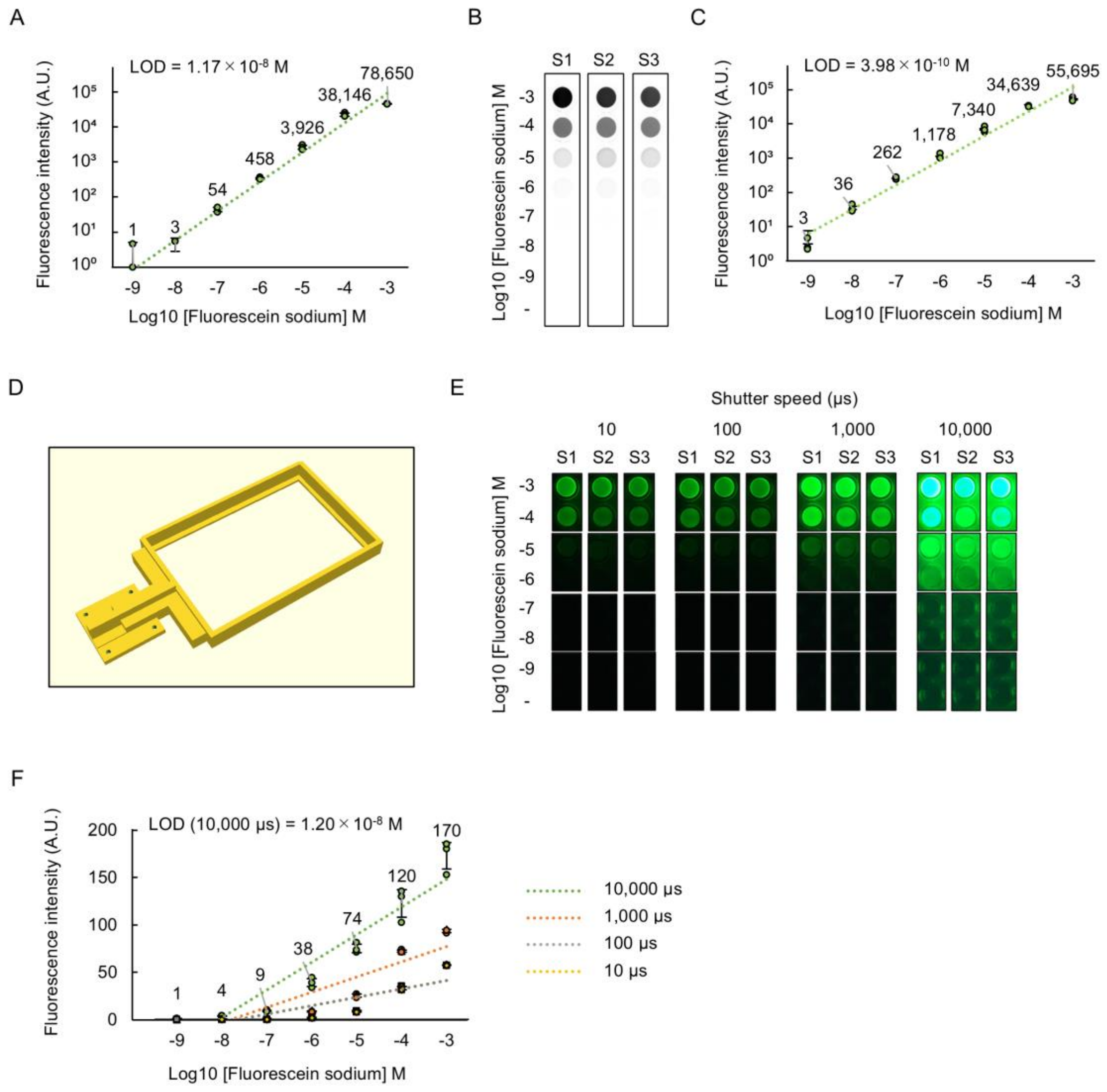

S6 Fig. Evaluation of fluorescence detection sensitivity using fluorescein and demonstration of expandability to plate reader. Sequential dilutions of a sodium fluorescein solution were poured in triplicates into wells of a 96-well plate to serve as standard samples (n=3). (A-C) The green fluorescence intensity for each concentration was measured using: (A) a plate reader (Mithras Berthold) and (B-C) a laser scanner (Amersham Typhoon). (B) Images of samples collected on the scanner and processed data (C). (D) To allow for comparisons with LOTUS, a plate sample tray was printed and attached to the system. (E) Fluorescence images were captured at different shutter speed and (F) the fluorescence intensity per well was measured. Individual data points are shown and error bars indicate SD (n=3).

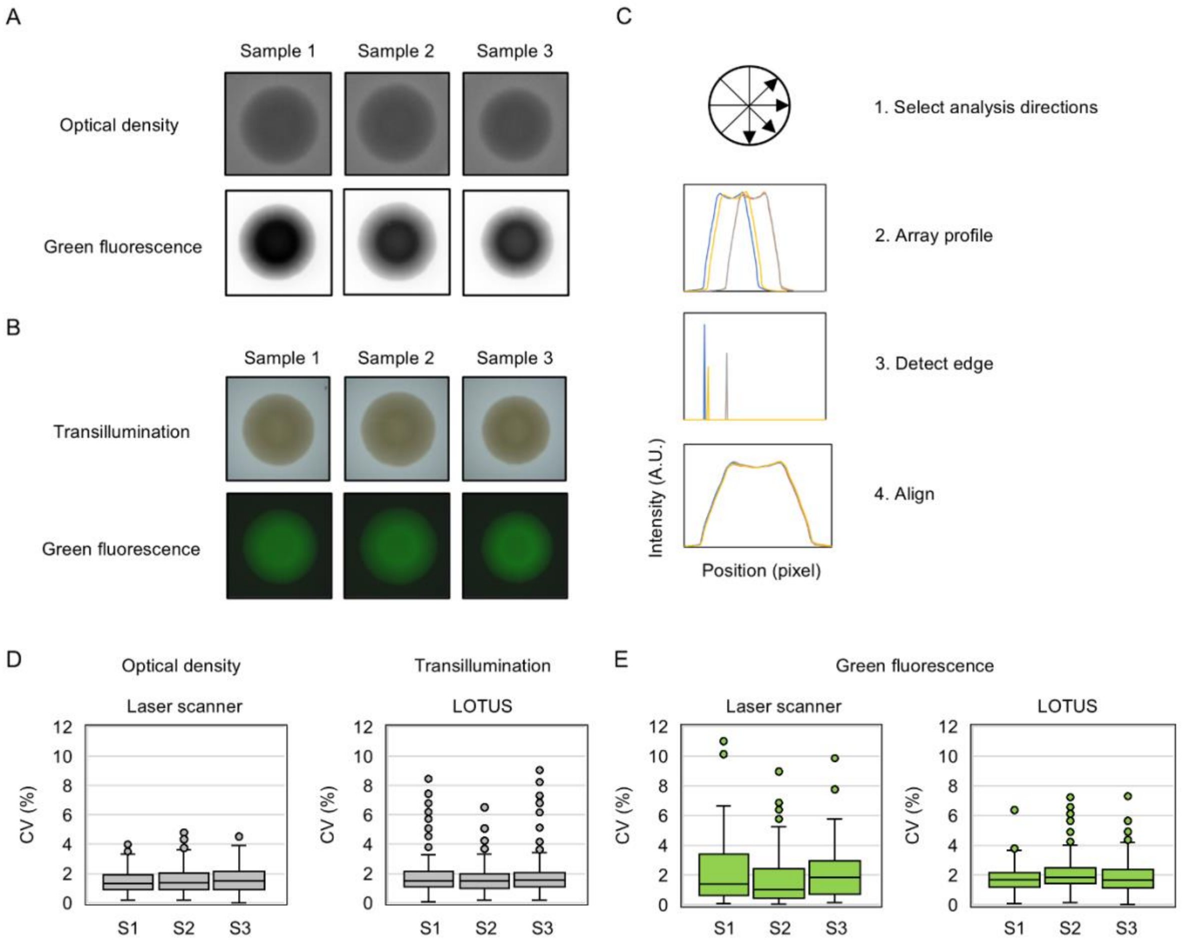

S7 Fig. Comparison of biofilm image profiles between LOTUS and a laser scanner. Triplicate biofilms were imaged on (A) a laser scanner or (B) LOTUS in bright-field (top) or fluorescence (bottom) modes. (C) Image profiles were analyzed in four directions and aligned as shown. Directional uniformity of signals is shown for all samples on both instruments for transillumination (D) and fluorescence (E) as CV. Box plots show the median, interquartile range (IQR) and whiskers extend to the most distant data point within 1.5 X IQR.

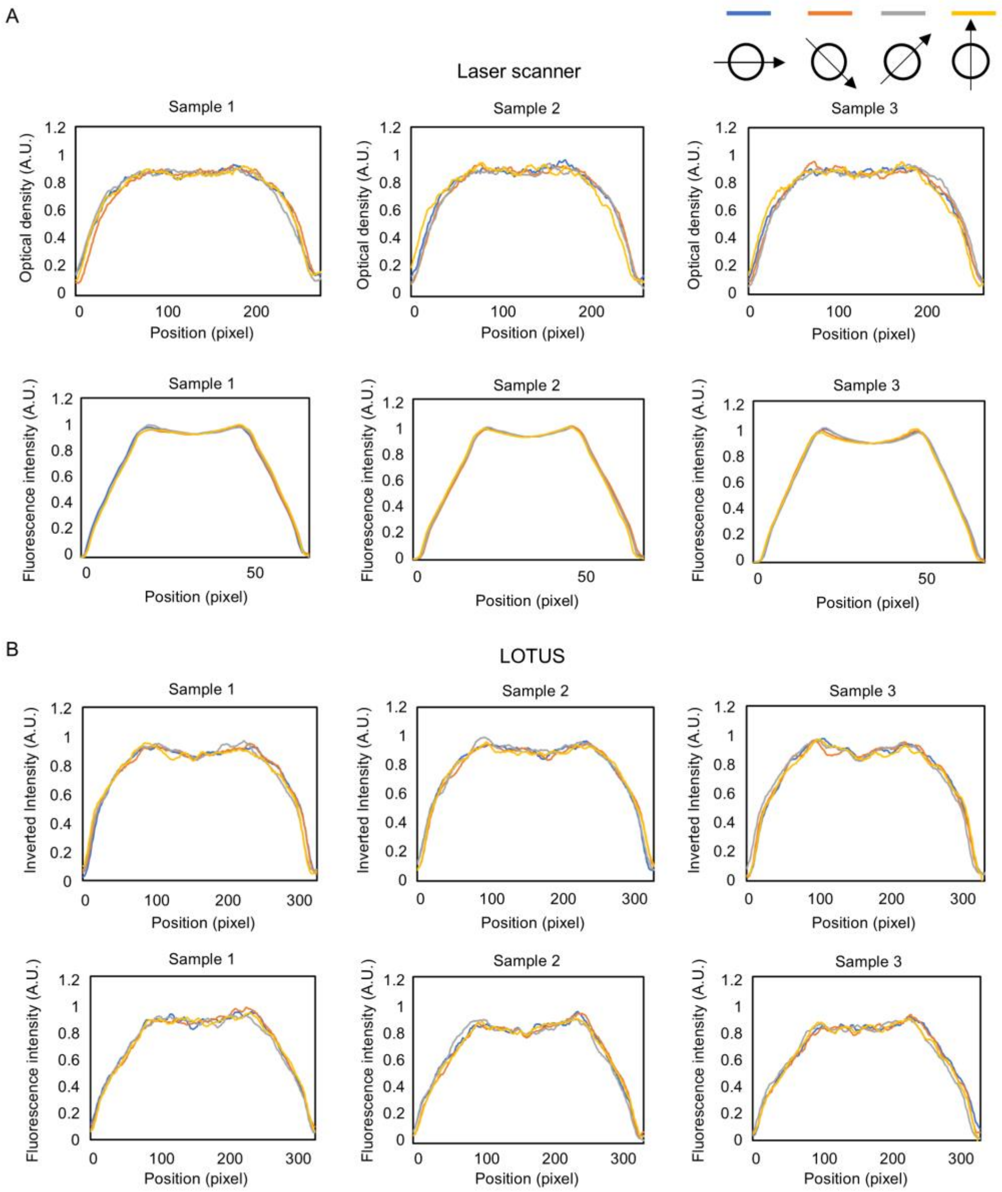

S8 Fig. Comparison of biofilm biomass and fluorescence profiles on laser scanner and LOTUS. Images of triplicate biofilm samples acquired using (A) a laser scanner and (B) LOTUS (S7 Fig) were analyzed in four directions (arrows) to generate intensity profiles for biomass proxy (transillumination, top) and fluorescence of the rsd promoter strain biofilms (bottom). In each plot, profiles from all four directions are overlayed.

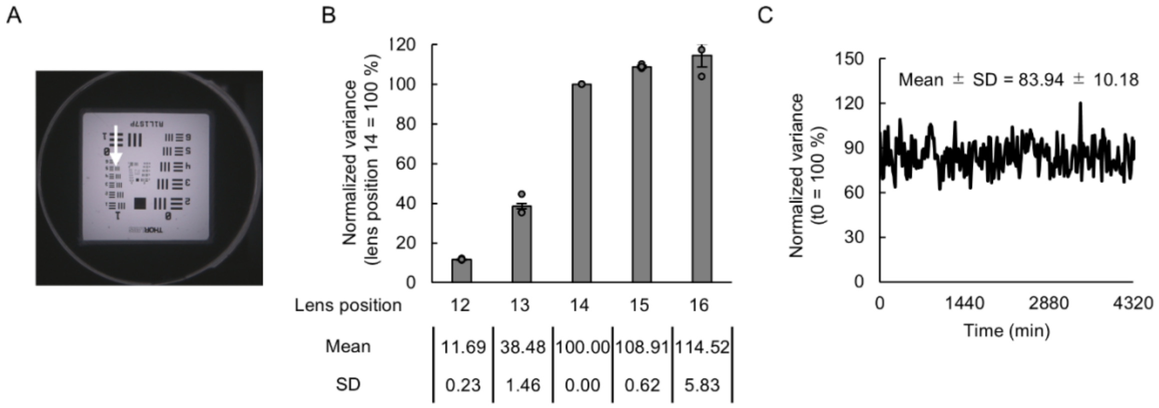

S9 Fig. Stability and reproducibility of focus. (A) View of the resolution target (B) Laplacian variance of Group 0 element 1 profile of the resolution target at different focus settings. Relative values are based on the lens position 14, used in this study. (C) Relative values of Laplacian variance based on initial images of resolution targets (Group 0 element 1), during three days of imaging.

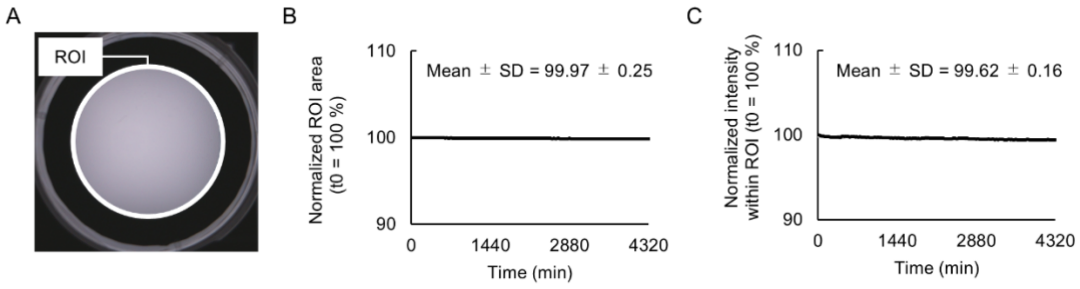

S10 Fig. Stability and reproducibility of area and intensity within ROI. (A) View of PLA disk with a 2.5 cm diameter opening placed in a 37mm dish. The white circle marks the ROI used for analysis. (B) Area and (C) intensity of the disk opening continuously imaged over 3 days.

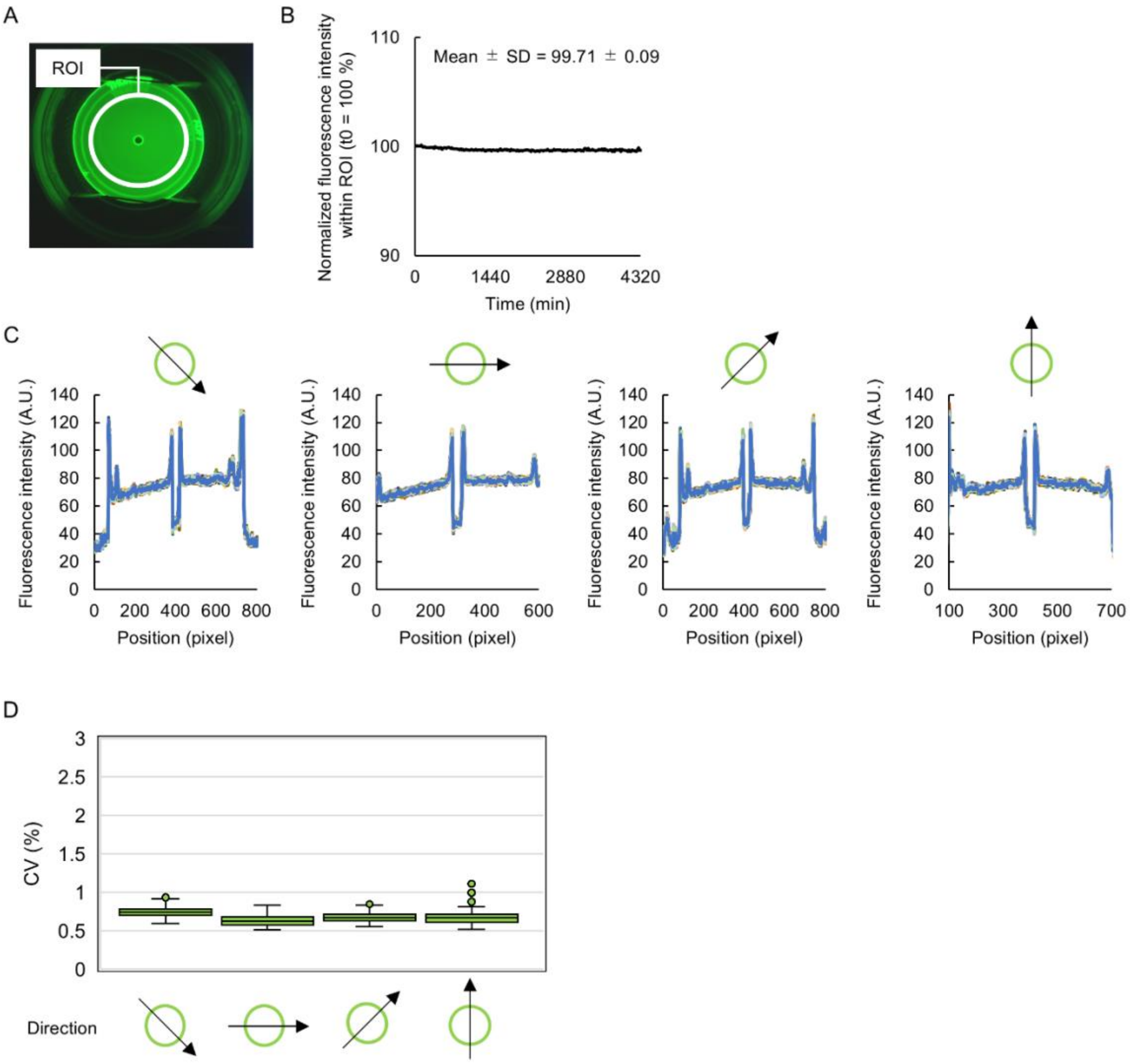

S11 Fig. Flatfield uniformity of fluorescence intensity and pixel intensity profile.

(A) Image of the green, fluorescent alignment disk placed in dish. ROI is marked by a white circle. (B) Mean ROI fluorescence intensity variation over time. (C) Fluorescence intensity profiles of the alignment disk analyzed from four directions. (D) Box-and-whisker plots showing spatial variation of fluorescence (CV) at each pixel position in each direction. Whiskers extend to the most distant data point within 1.5 X IQR.

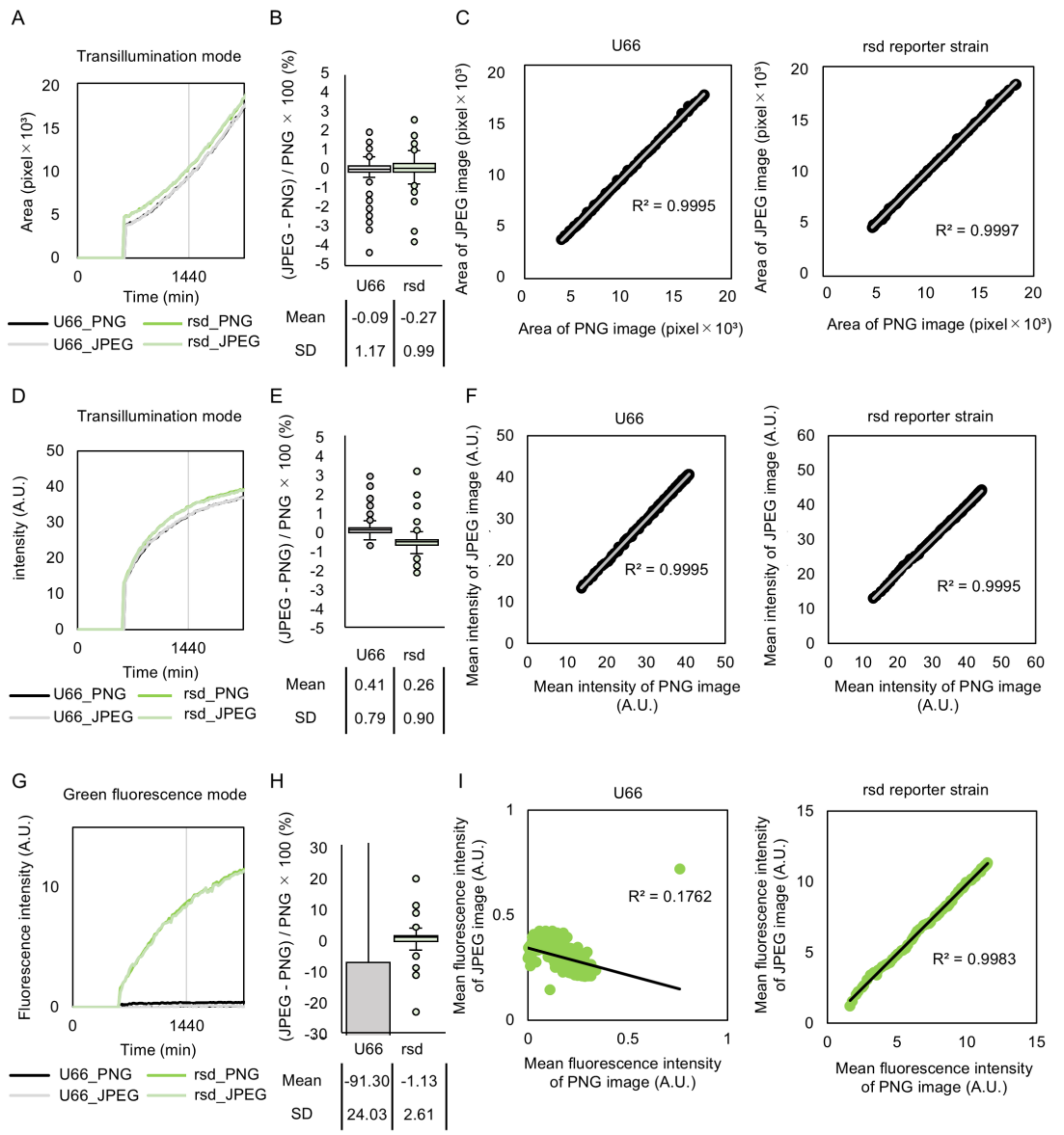

S12 Fig. Analysis of quantitative effects of image compression. Temporal changes in (A) biofilm area, (D) transmitted light intensity, and (G) fluorescence intensity acquired from PNG and JPEG images. (B, E, and H) Box-and-whisker plots showing the relative difference between JPEG and PNG values, calculated as (JPEG − PNG) / PNG × 100 (%) for panels A, D, and G, respectively. (C, F and I) Scatter plots showing PNG values plotted against JPEG values using measurements from the U66 and rsd reporter strains for panels A, D, and G, respectively.

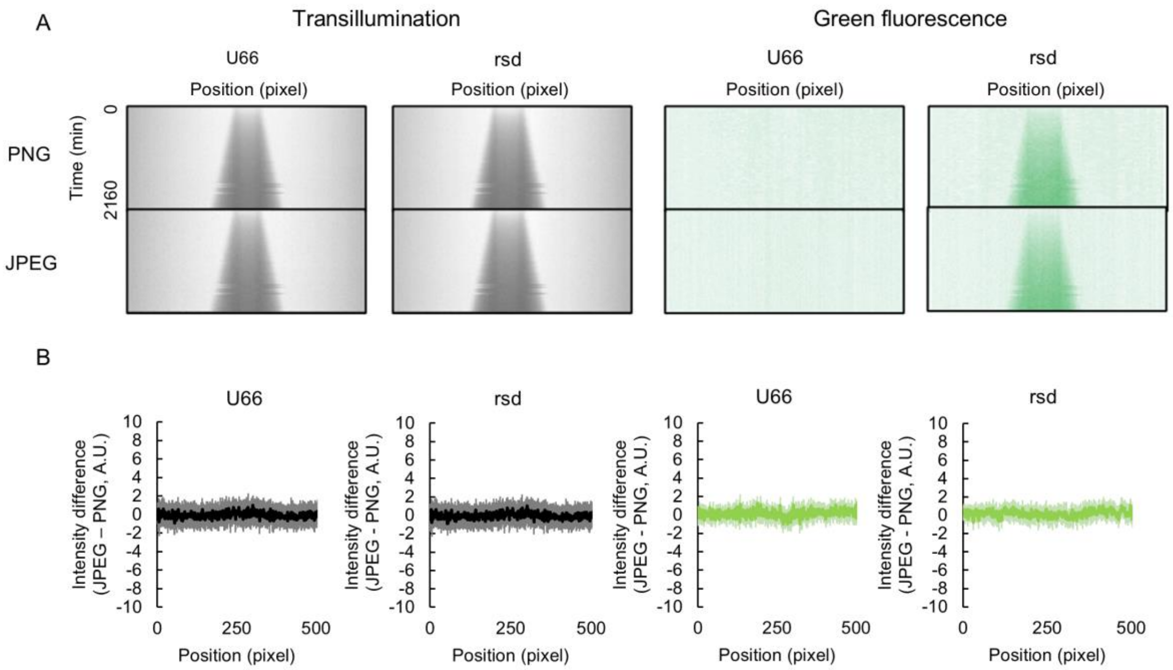

S13 Fig. Analysis of differences in kymographs between PNG and JPEG images. (A) Kymographs generated from both PNG and JPEG image sets for the indicated promoters and imaging modes. (B) Mean absolute pixel intensity difference between JPEG image and PNG images over a full imaging cycle.

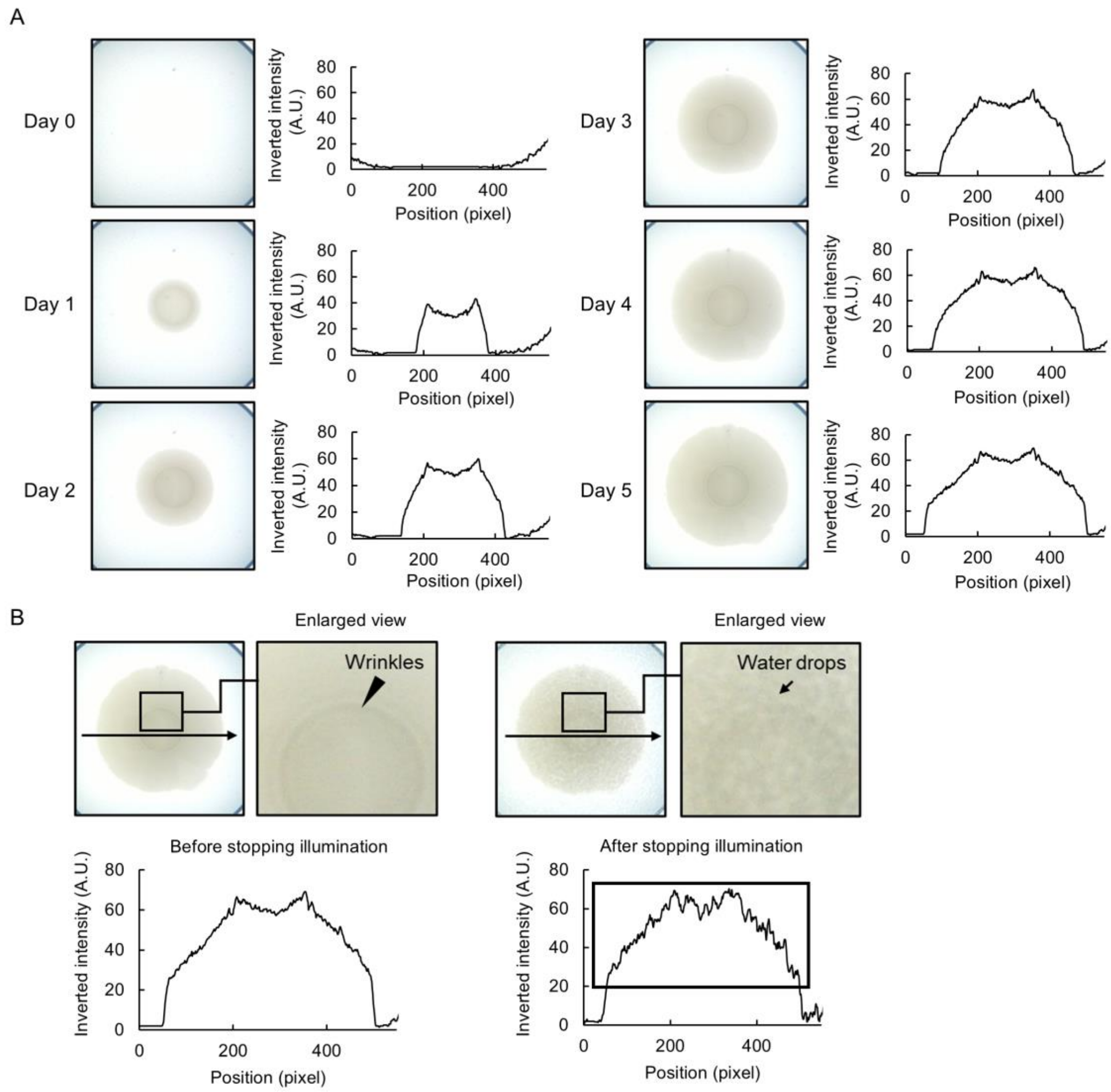

S14 Fig. Lack of apparent condensation during imaging. (A) Transillumination images and pixel intensity profile of a growing biofilm over 5 days. (B) Close up view of biofilm and pixel intensity profiles of biofilm images taken immediately at the end of five days of imaging (left) and 30 min after imaging ended (right). Arrows drawn through the center of the biofilm indicate the direction of image analysis.

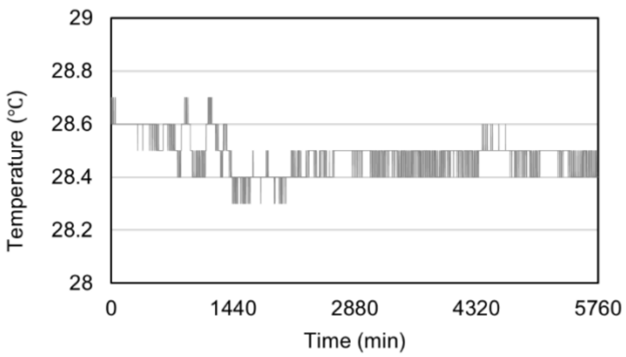

S15 Fig. Fluctuations in temperature during operation. A temperature sensor was positioned in the incubator and temperature was measured over four days of system operation.

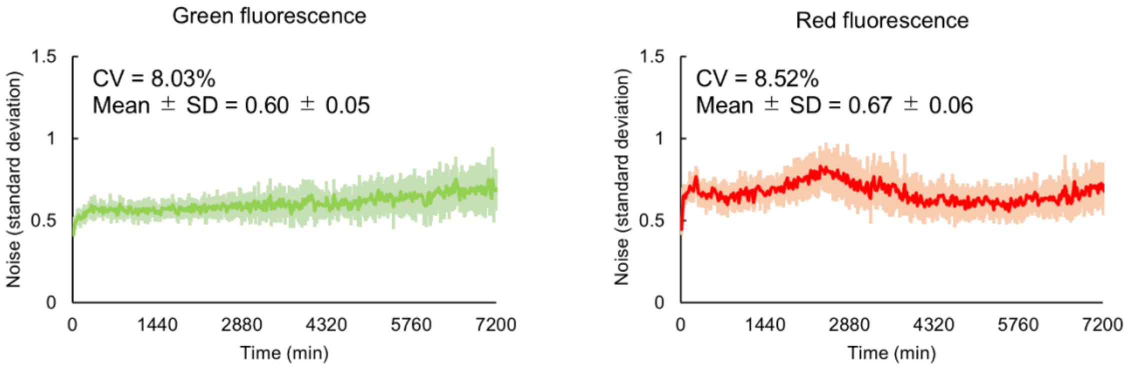

S16 Fig. Temporal quantification of noise of fluorescence image. Mean noise was calculated using the standard deviation of fluorescence intensity of the area outside the identified biofilm edges. The lines in the graph represent the mean values across datasets, while the shading indicates the standard deviation (n=9, technical replicates).

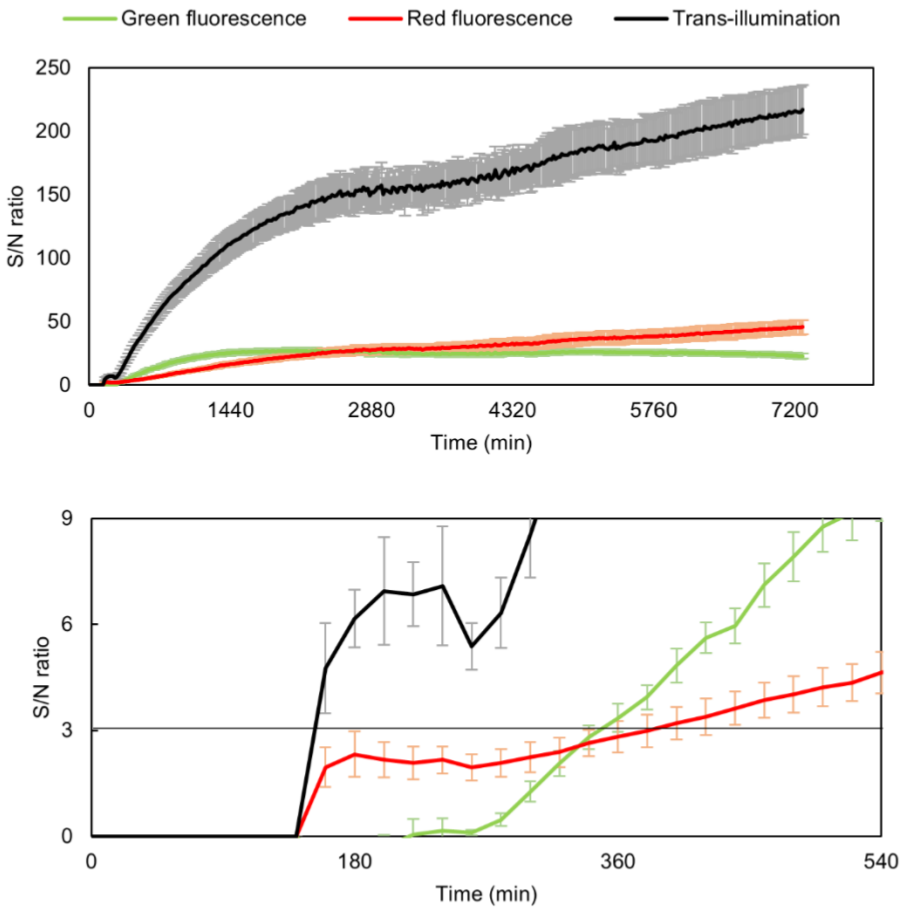

S17 Fig. Temporal quantification of S/N ratio. The standard deviation of the fluorescence intensity in the region outside the biofilm edge (ROI) was used as noise, and the mean fluorescence intensity detected in the region inside the biofilm edge was used as the signal. The upper graph shows the variation in S/N ratio for all wells over the whole experiment, while the lower graph shows the same variation during the first 540 min. The lines represent the mean value and error bars indicate the standard deviation (n = 9, technical replicates).

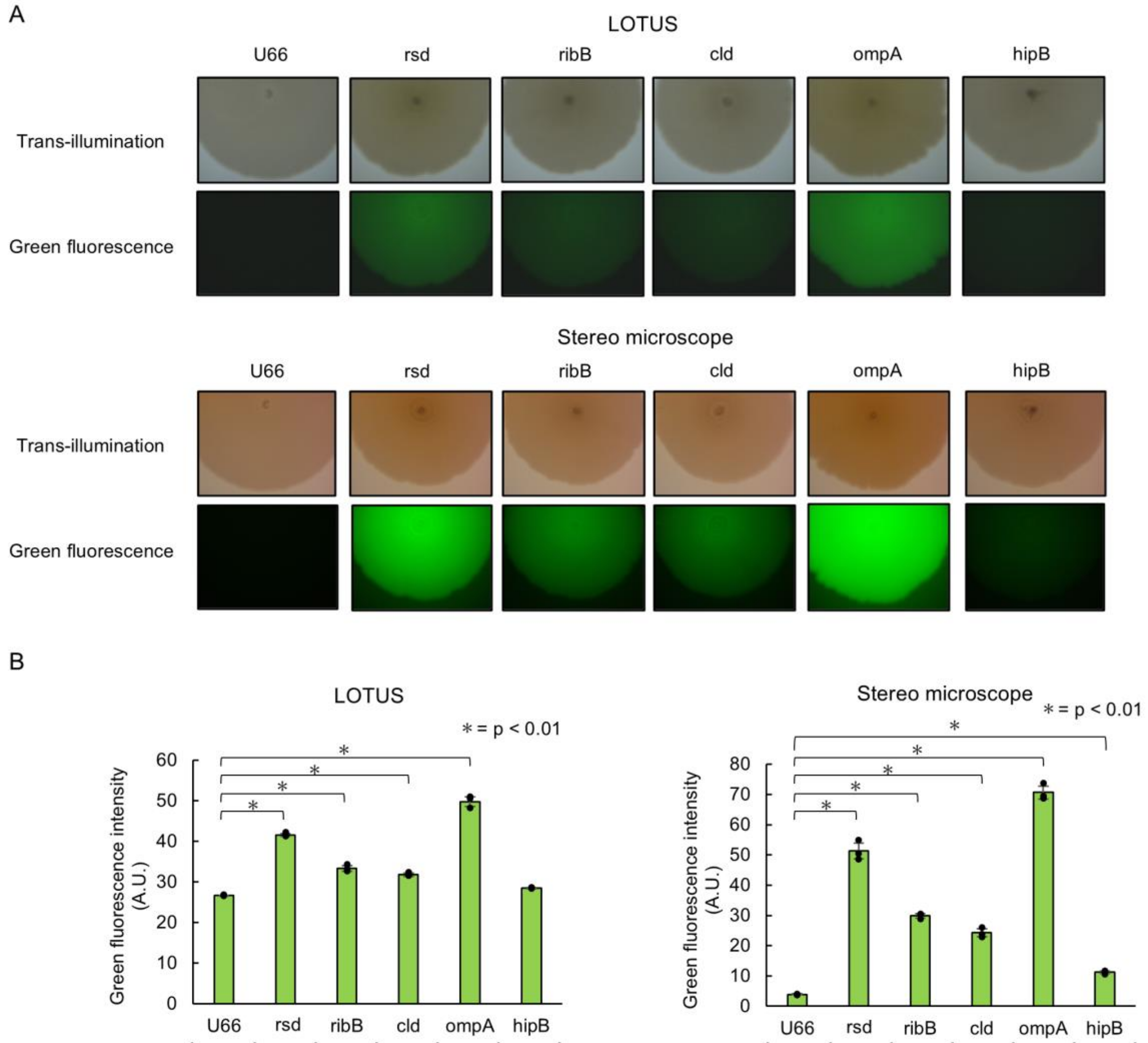

LOTUS

	U66	rsd	ribB	cld	ompA	hipB
Mean	26.6	41.6	33.3	31.8	49.8	28.4
SD	0.0	0.4	0.7	0.4	1.2	0.1

Stereo microscope

	U66	rsd	ribB	cld	ompA	hipB
Mean	3.9	51.3	29.9	24.3	70.6	11.2
SD	0.2	2.7	0.7	1.3	2.2	0.4

S18 Fig. Comparison of fluorescence intensities between LOTUS and stereomicroscope. (A) Transillumination images and green fluorescence images of different promoter strains imaged with LOTUS and a stereomicroscope. (B) Fluorescence intensity in the biofilms of each promoter strain for both systems. Bars show mean value and error bars indicate SD (n=3, technical replicates).

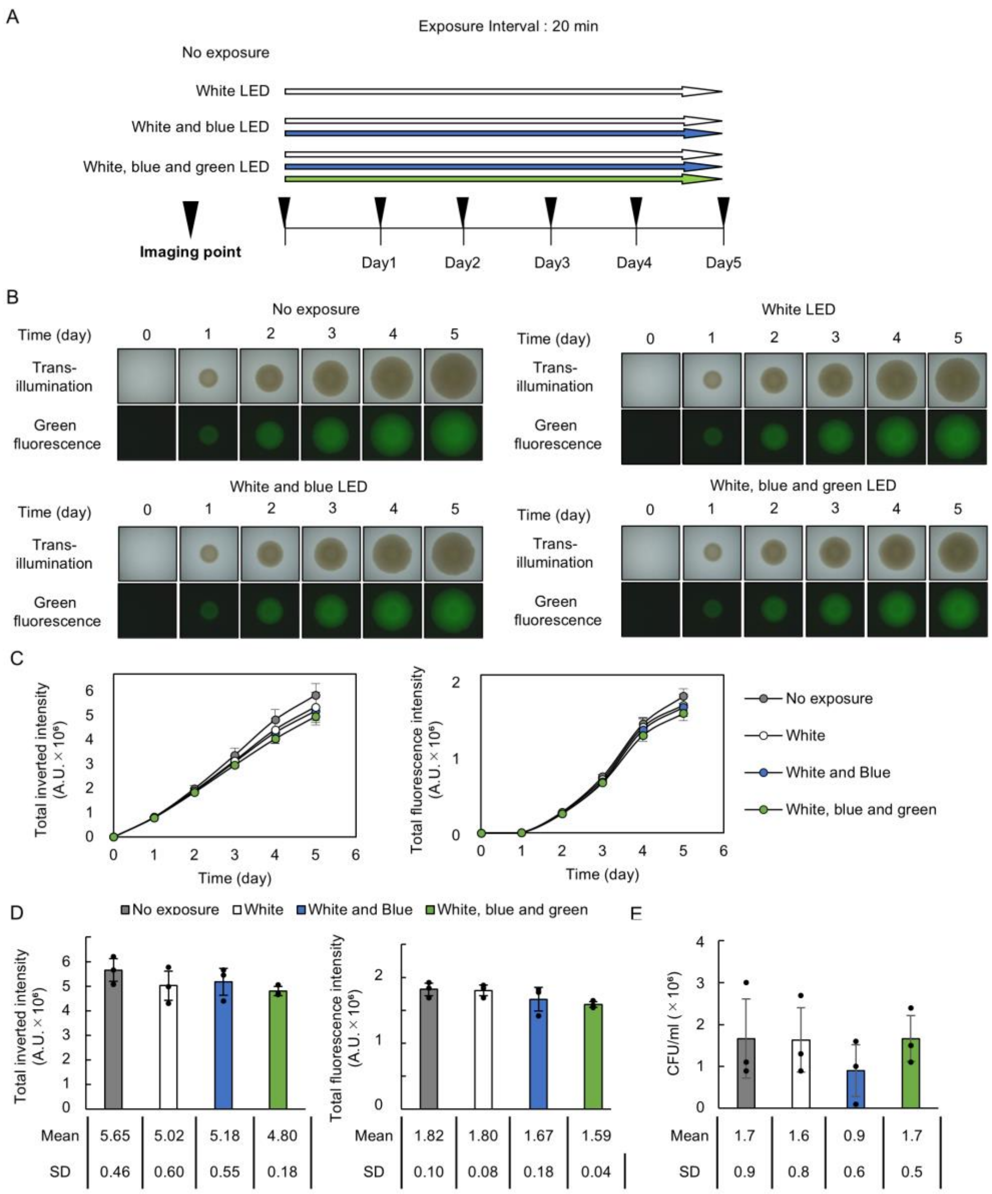

S19 Fig. Evaluation of phototoxicity. (A) Biofilms of the rsd promoter strain were imaged under various exposure conditions. Control biofilms were unexposed to LED illumination except at specific imaging time points (once every 24 h) shown by arrow heads. Biofilms were illuminated every 20 min in only transillumination (White LED), transillumination and blue epi-illumination (White and blue LED) or transillumination, blue and green epi-illumination (White, blue and green LED). The LED exposure time for each source was the same as used for routine imaging, around 8 to 9 sec. (B) Transillumination and green fluorescence images of rsd promoter strain biofilms imaged once a day under each exposure condition. (C) Biomass proxy (inverted intensity) and green fluorescence intensity signals during growth of biofilms under each exposure condition. Error bars indicate SD. (D) Total biomass proxy (inverted

intensity) and green fluorescence intensity of biofilms at the end point for each exposure condition. (E) CFU/ml of the whole biofilms collected at the end point. Bars show mean value and error bars indicate SD (n=3, biological replicates).

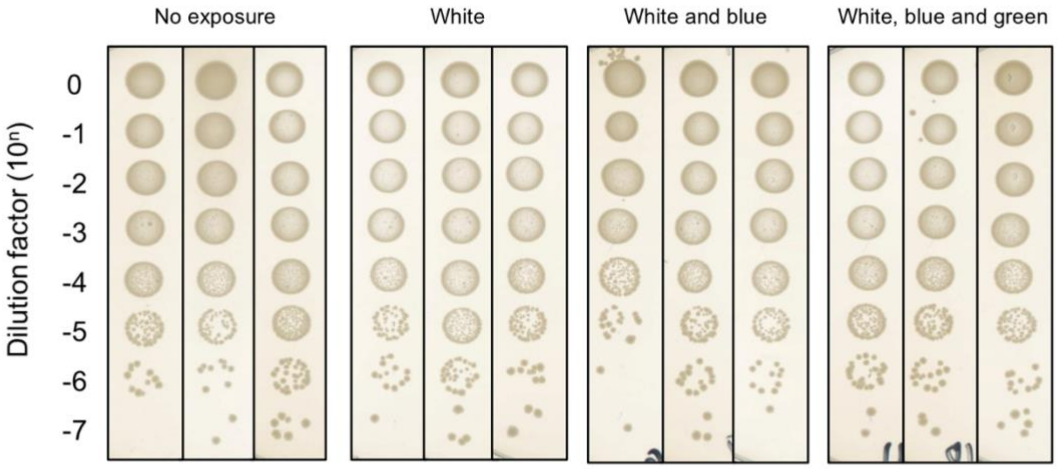

S20 Fig. Cell viability following different light exposure protocols. Biofilms were sampled from agar, suspended in PBS, serially diluted, and 10 µl was spotted onto LB agar plates and incubated at 37°C for 18 hours. CFUs were counted manually. More details in Supporting Methods.

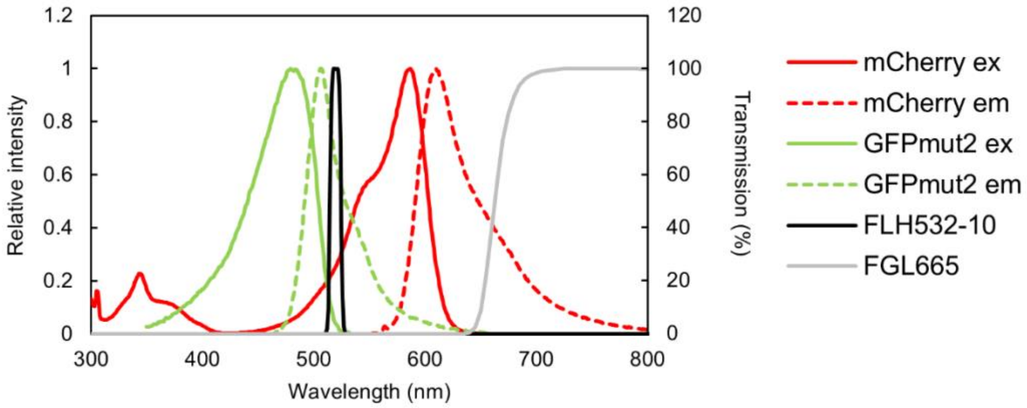

S21 Fig. Emission and excitation spectrum of GFPmut2 and mCherry and transmission properties of optical filters. The green and red lines show the excitation (solid line) and emission (dashed line) spectra of GFPmut2 and mCherry, respectively. The black and grey lines indicate the wavelengths transmitted by each filter. Spectrum data were obtained from FPbase (The Fluorescent Protein Database).
