# Supplementary material for: LOTUS: A low-cost time-lapse automated imaging system for spatio-temporal analysis of microbial colony or biofilm development
Source: PLoS One. 2026 Jan 23;21(1):e0339652. doi: 10.1371/journal.pone.0339652 (PMC12829848; doi:10.1371/journal.pone.0339652)
Supplement: S2 File — (PDF) [file pone.0339652.s002.pdf]

Supporting tables

S1 Table. Overview of total costs for LOTUS development

Prices are approximate and may vary by supplier and region.

Total cost ($)

Resource	Supplier	Unit price ($)	Quantity	Total price ($)
100 Ω resistor	uxcell	0.01	20	0.20
18V power	Kaito Denshi	5.91	1	5.91
5V power	Aifulo	6.56	1	6.56
64 MP camera	Arducam	65.64	1	65.64
Acrylic plate 420 × 297 × 3 mm	SUMIKA ACRYL	10.83	1	10.83
Circuit board (ICB288GU)	Sanhayato	1.46	4	5.84
Bread board	Sanhayato	0.98	1	0.98
Connector pin and housing set	Bttime	6.56	1	6.56
DC jack	uxcell	0.53	2	1.06
Double-sided tape	3M	3.94	1	3.94
Expansion board	KEYESTUDIO	6.56	1	6.56
Grease	AZ	6.56	1	6.56
Jumper wire set (20 cm)	Techcell	4.92	2	9.84
Jumper wire set (roll)	neshxt	13.13	2	26.26
LEDs (White, blue, and green)	LED paradise	0.26	100	26
Optical filter (510-530 nm band pass filter)	Thorlabs	164.1	1	164.10
Optical filter (665 nm long pass filter)	Thorlabs	32.82	1	32.82
Pin header kit	Wayintop	9.19	1	9.19
PLA filament 1 kg (black)	Creality	13.13	1	13.13
Raspberry pi 4 model B	Raspberry pi	91.89	1	91.89
Relay modules	Wayintop	0.72	4	2.88
Flux-cored solder wire Tin/Lead 60/40	goot	15.1	1	15.10
M3 screw kit	HGPJLEE	29.54	1	29.54
Stepping motors and driver (28BYJ-48 and ULN2003)	VKLSVAN	1.97	5	9.85
Total cost ($)				551.24
**USD/JPY＝152.35**				

To build the system, we used the following 3D printer:

3D printer Creality Ender3 s1	Creality	210.04	1	210.04

S2 Table. Imaging operation cycle

Imaging cycle process	Duration (s)
**Movement of sample stage**	
**Movement positions (number of steps = x-axis steps + y-axis steps)**	
home to well 1 (steps=480+130)	8.9
well 1 to 2 (steps=600)	8.8
well 2 to 3 (Steps=600)	8.8
well 3 to x axis home (Steps=1780)	26.0
home to well 1 (steps=480)	7.0
well 1 to 4 (Steps=600)	8.8
well 4 to 5 (Steps=600)	8.8
well 5 to 6 (Steps=600)	8.8
well 6 to x axis home (Steps=1780)	26.0
home to well 4 (steps=480)	7.0
well 4 to 7 (Steps=600)	8.8
well 7 to 8 (Steps=600)	8.8
well 8 to 9 (Steps=600)	8.8
well 9 to home (steps=1780+1320)	45.2
**Imaging process (same for each well)**	
**Transillumination imaging**	
Turning the white LED array (trans-illumination) ON	3.0
Capture image	8.0
Turning the white LED array (trans-illumination) OFF	3.0
**Green epi-fluorescence imaging**	
Rotate the filter wheel and illumination module from 0 to 90° (Steps=128)	1.9
Turning the blue LED array (epi-illumination) ON	3.0
Capture image	8.0
Turning the blue LED array (epi-illumination) OFF	3.0
**Red epi-fluorescence imaging**	
Rotate the filter wheel and illumination module from 90 to 180° (Steps=128)	1.9
Turning the green LED array (epi-illumination) ON	3.0
Capture image	8.0
Turning the green LED array (epi-illumination) OFF	5.0
**White epi-illumination imaging**	
Rotate the filter wheel and illumination module from 180 to 0° (Steps=256)	3.7
Turning the white LED array (epi-illumination) ON	3.0
Capture image	8.0
Turning the white LED array (epi-illumination) OFF	0.0
**Total cycle time**	752.2
Time (s) / step (by calibration)	0.0146

S3 Table. Camera settings

Raspberry Pi OS (Operating System: Raspbian GNU/Linux 11 bullseye, Kernel: Linux 6.1.21-v8+ Architecture: arm64 released 2024/03/12)
The camera module was configured as described in Arducam wiki (https://docs.arducam.com/Raspberry-Pi-Camera/Native-camera/Quick-Start-Guide).

**imaging parameters in camera (64 mp Hawkeye)**	
**transillumination and epi-illumination white image**	
image resolution	2312 × 1736
contrast	1
brightness	0.1
shutter	500
denoise	off
autofocus mode manual	lens position 14
gain	2

**Green fluorescence image**	
image resolution	2312 × 1736
contrast	1
brightness	0.1
shutter	3000
denoise	off
autofocus mode manual	lens position 14
gain	2
awgains	1.5, 2.0

**Red fluorescence image**	
image resolution	2312 × 1736
contrast	1
brightness	0.1
shutter	30000
denoise	off
autofocus mode manual	lens position 14
gain	2
awgains	1.5, 2.0

**Measurement parameters of plate reader (Mithras LB940)**	
Plate type	96-well plate
Counting time [s]	0.05
Measurement mode	by plate
Excitation filter	F485
Emission filter	F535
Excitation aperture	Normal
Lamp Energy	1000
Counter Position	Top

**Imaging parameter in laser scanner (Amersham Typhoon)**	
Laser name	488 nm
Filter name	Cy2 525BP20
PMT Type	Multi-alkali
Voltage	250
Laser power mode	High
Pixel size (μm / pixel)	200

S4 Table. Basic comparison with commercial systems

Feature	LOTUS	Entry-level commercial	High-end systems (automated fluorescence microscope)
Cost	~$550 USD	$8,000-15,000	>$50,000
Resolution	0.14 mm	0.05-0.1 mm	<0.01 mm
Samples/run	9 (expandable to 12)	1-24	96+
Fluorescence channels	2 (expandable)	1-2	3-5
Automation	Full	Partial	Full
Customization	High	Low	Medium
