# Supplementary material for: LOTUS: A low-cost time-lapse automated imaging system for spatio-temporal analysis of microbial colony or biofilm development
Source: PLoS One. 2026 Jan 23;21(1):e0339652. doi: 10.1371/journal.pone.0339652 (PMC12829848; doi:10.1371/journal.pone.0339652)
Supplement: S3 File — (PDF) [file pone.0339652.s003.pdf]

Supporting Information (Sakai et al.)

LOTUS: A Low-cost time-lapse automated imaging system for spatiotemporal analysis of microbial colony or biofilm development

CONTENTS

SECTION 1: System information

System version, assembly, software, cost breakdown, imaging cycle, camera settings

SECTION 2: Supporting methods

Competent cell preparation and transformation
Cell viability quantification

SECTION 3: Validation and technical characterization

Stability during time-lapse imaging (focus, area, intensity, fluorescence)
Image compression effects on quantitative measurements
Temperature fluctuations and condensation
Phototoxicity evaluation

SECTION 4: Technical details and design considerations

Well position alignment • Resolution • LED specifications • Controller specifications
Noise and signal-to-noise ratio • Fluorescence inhomogeneity
Hardware degradation • Customizability and sample format adaptation

SECTION 5: Measurement considerations and interpretation

Biomass measurements using transillumination
Fluorescence measurements and interpretation
Sensitivity estimation for GFP detection

SECTION 6: Comparison with other imaging systems

Open-source systems (FlyPi, Picroscope, Futo et al., French et al., Nuñez et al., OpenIVIS)
Commercial systems (Celloger Nano)

SECTION 7: Data availability

Section 1: System information

LOTUS system version

We refer to this first version of the imaging system as LOTUS-9 v1.0 (9-well version). We also have developed a 12-well sample tray version that is otherwise identical and named LOTUS-12 v1.0. Future versions with alternative number of samples can be named accordingly.

System assembly

LOTUS can be assembled and operational within 2-3 days for users with basic experience in 3D printing and electronics, following the instructions herein. Assembly videos are also available on our GitHub repository (see Data availability).

Software versions

The following software versions were used in this study:

- Fiji/ImageJ: Version 1.53c or later
- Python: Version 3.8 or later (for image processing scripts)
- Raspberry Pi OS: Bullseye (2024-03-12 release)
- OpenSCAD: Version 2021.01
- FreeCAD: Version 0.21.2
- jamovi: Version 2.7.12.0
- IrfanView version 4.62

System cost breakdown

See S1 Table for detailed component costs totaling approximately $550 USD.

Imaging cycle details

See S2 Table for complete imaging cycle operation sequence (approximately 12 minutes per 9-well cycle).

Camera settings

See S3 Table for complete camera parameters and image acquisition settings.

Section 2: Supporting methods

Preparation of competent cells and transformation

Competent cells were prepared using the calcium chloride method [1]. Specifically, bacterial glycerol stocks stored at -80°C were streaked on LB agar plates and incubated for 16 h at 37°C. A single colony was inoculated into 2 ml of liquid LB medium and incubated for 16 h in a shaker at 37°C and 250 rpm. Then, 20 µl of overnight cultures were inoculated into 2 ml of LB liquid medium and incubated at 37°C for 2 hours at 250 rpm. The cultures were then washed twice with 50 mM calcium chloride solution on ice to prepare chemical competent cells. Plasmid purification was performed using the QIAprep Spin Miniprep Kit (Qiagen). A volume of 5 µl of plasmid solution was added to 25 µl of chemical competent cells and the cells were incubated on ice for 30 min, heat shocked at 42°C for 1 min, and then incubated on ice for another 2 min. A volume of 270 µl of LB medium was added to each transformant and incubated at 37°C for 1 hour with shaking at 250 rpm. Finally, 100 µl of the transformed cells were inoculated onto solid LB medium supplemented with antibiotics (ampicillin 100 µg/ml and kanamycin 25 µg/ml). Plates were incubated overnight (approximately 12-16 hours) at 37°C for colony formation.

References

1. https://asm.org/asm/media/protocol-images/transformation-of-escherichia-coli-made-competent-by-calcium-chloride-protocol.pdf?ext=.pdf

Quantification of cell viability

To quantify viable cells within biofilms, the entire agar piece containing the surface-attached biofilm was excised and transferred into a sterile 50 ml conical tube containing 8 ml of sterile phosphate-buffered saline (PBS) adjusted to pH 7.4. The agar and attached biofilm were then vigorously vortexed for 30 seconds to dislodge and suspend bacterial cells into the PBS solution. The resulting suspension was

serially diluted 10-fold into PBS, and 10 μl of all dilutions were spotted onto LB agar plates. Plates were incubated at 37°C for 16–18 hours, and colony-forming units (CFUs) were counted manually.

Section 3: Validation and technical characterization

Focus stability

The Laplacian variance is a simple and commonly used test to quantify image sharpness (focus). To quantify possible focus variation over time, images of the resolution target were taken while changing the lens position range (14 ± 2), and the Laplacian variance at Group 0 Element 1 was calculated (S9A-B Fig). The Laplacian variance decreased sharply when the lens position was changed from 14 to 12 and showed a slight increase when changed from 14 to 16. The mean value was approximately 38% at lens position 13, 100% at lens position 14 (focus set), and about 115% at lens position 16. Next, the resolution target was imaged over three days and the Thorlab resolution target Group 0 Element 1 lines were analyzed. Normalized Laplacian variance values collected over the three days are shown in S9C Fig). The results showed temporal Laplacian variance fluctuations in the 60-120% range (mean 83.9%), compared to < 40% after changing the lens position setting to 13 or below. While focus drift during time-lapse imaging could not be ruled out, it appears negligible under the conditions used and other imaging parameters were stable (next section).

Area and intensity stability

We conducted tests to confirm whether imaging area or intensity fluctuates during continuous imaging of the same non-biological sample. First, a PLA disk with a 25 mm diameter circular hole was created using a 3D printer and imaged in white transillumination mode (S10A Fig). Variation in area and mean intensity within the PLA disk were then measured (S10B-C Fig). The results show that the measured normalized area and mean intensity within the PLA disk hole did not change over time (area: 99.97 ± 0.25%, intensity: 99.62 ± 0.16%). These results thus confirm the stability of illumination during transillumination imaging.

Fluorescence intensity stability

Next, to confirm whether the same applies in fluorescence mode, a green fluorescence disc was imaged over time (S11A Fig) and variation in fluorescence intensity at the center region of the disk was

measured. The results showed that fluorescence intensity remained unchanged (mean fluorescence intensity 99.71 ± 0.09, S11B Fig). In addition, as in Fig 6, spatial profiles were obtained by measuring intensity through the center of the disk along four directions (S11C Fig). Per pixel CV was then calculated using all profiles in each direction, and plotted (S11D Fig). The CV was below 1.25% for all measurements confirming that there were no significant fluctuations in spatial fluorescence intensity over a 3-day imaging period.

Effect of JPEG image compression on quantitative measurements

Due to current hardware limitations, the Raspberry Pi computer was set to capture images in JPEG format. Because image capture in JPEG format results in lossy compression and smoothens local intensity variations, this can affect the results of quantitative image analysis at fine spatial scales. Therefore, we investigated how converting PNG images acquired with LOTUS to JPEG affected biofilm area, inverted intensity, fluorescence intensity and spatial profiles (kymographs). To do this, we acquired time-lapse datasets from growing biofilms (U66 and rsd reporter strains) in PNG format. Images were converted to JPEG using IrfanView with Save quality: 80 and other settings as default.

JPEG compression caused negligible variation in the ROI area (r-squared value > 0.99) (S12 Fig) and mean intensity value (< 5% relative difference) of transillumination. Results for fluorescence were affected for very low or near background fluorescence levels (U66 strain) but not for higher levels. In addition, the compression had little effect on spatial signals as in kymographs and the intensity difference between PNG and JPEG files was within ± 2 for the promoterless strain and rsd promoter, for all pixel positions (S13 Fig).

Comparative analysis of PNG vs JPEG formats revealed that compression artifacts introduced quantitative errors of approximately 5%. This level of error is acceptable for semi-quantitative analysis across multiple samples and large ROIs but may affect detailed quantitative comparison between adjacent pixels (S12 Fig and S13 Fig). We recommend uncompressed standard formats (TIFF, PNG) for precise quantitative applications.

Temperature fluctuations and condensation

To promote biofilm formation, *E. coli* is often incubated below 30°C [1-4] and in this study, we grew biofilms at 28°C. Since biofilm morphogenesis is affected by temperature and humidity, it is important to maintain a constant temperature and humidity in the incubation environment to guarantee reproducibility. When imaging biofilms over time, the sample temperature may rise temporarily due to the proximity of the LEDs and moisture from the agar medium can condense on the plate lid, causing blurred images (S14 Fig). One solution to avoid this problem was developed by Futo and colleagues, using a simple robotic arm that automatically removes the lid of the Petri dish for imaging [5]. However, opening and closing the lid repeatedly over long periods may cause contamination of samples and the incubator.

To maintain humidity and prevent agar dehydration, sample dishes were sealed with Parafilm and placed upside down on the system sample tray and biofilm development was captured using transillumination every 20 minutes for 120 hours (5 days). To intentionally cause condensation, at the end of the imaging cycle one Petri dish was removed from the tray, left in the incubator for 5 minutes, then imaged and analyzed in the same manner (S14B Fig). Incubator temperature fluctuations were recorded every minute using a Govee thermometer (S15 Fig).

To evaluate whether condensation on the lid of the Petri dish was problematic, signal intensity was measured along the line crossing the biofilm center each day (S14B Fig). As seen on the profiles of the bright-field image taken immediately after 5 days of incubation, transmitted light intensity tended to decrease toward the high-density area in the center or the outer edge of the biofilm. The profile became more jagged for the sample left in the incubator for 30 mins after stopping the imaging due to water droplet condensation (arrowheads, framed area S14 Fig C). These results suggest that periodical illumination reduced temperature gradients around the Petri dish lid, preventing significant moisture condensation. Although not evaluated, condensation might be more likely to occur when using longer imaging interval. Only small variations in incubator temperature (28.3°C to 28.7°C) were measured during systems operation (S15 Fig) and since the incubator used does not have a cooling function, there is no sign of significant heat accumulation during the imaging period.

References

1. Serra DO, Richter AM, Hengge R. J Bacteriol. 2013 Dec;195(24):5540–5424.

2. Serra DO, Richter AM, Klauck G, Mika F, Hengge R. mBio. 2013 Feb;4(2):e00103-13.

3. Serra DO, Klauck G, Hengge R. Environ Microbiol. 2015 Dec;17(12):5073–88.

4. Weber H, Pesavento C, Possling A, Tischendorf G, Hengge R. Mol Microbiol. 2006;62(4):1014–34.

5. Futo M, Široki T, Koska S, Čorak N, Tušar A, Domazet-Lošo M, et al. Sci Rep. 2022 Dec; 7;12(1):21120.

Evaluation of phototoxicity during time-lapse imaging

LOTUS is designed for time-lapse observation, but prolonged exposure may affect bacterial gene expression and survival rates [1]. Therefore, in this section, we evaluated changes in biofilm morphology and the number of surviving cells associated with time-dependent exposure.

To evaluate the effects of LED exposure over time, we conducted experiments using reporter strains with genetically engineered reporter genes under the following conditions: 1) no exposure; 2) white LED exposure; 3) white LED exposure and blue LED exposure; 4) white LED exposure, blue LED exposure, and green LED exposure.Exposure to light was every 20 minutes for five days (S3 Video) (S19 Fig). Additionally, white transillumination images and green fluorescence images were acquired each day for all samples (S19A Fig). Fluorescence intensity detected from biofilms at each time point was plotted as cell density vs time, along with inverted intensity (biomass proxy, S19B-C Fig). Furthermore, inverted intensity and fluorescence intensity between the non-exposed and exposed conditions were measured and analyzed on day 5 (S19D Fig). Finally, the biofilm on day 5 was collected and suspension was diluted with PBS. The diluted solution was spotted onto agar medium and incubated at 37°C overnight (S20 Fig). Colonies were counted, and CFU was calculated based on the dilution ratio. Additionally, Welch's t-test was performed on the CFU/ml values between the non-exposed and exposed conditions ($p < 0.05$, Welch's t-test) (S19D Fig).

No characteristic biofilm morphological or fluorescence pattern changes were observed following any exposure conditions (S19B Fig). The inverted pixel intensity (biomass proxy) of white transillumination and the green fluorescence intensity in exposed samples was slightly, but not significantly lower than for non-exposed samples and no significant differences in CFU counts were observed among all conditions (p

> 0.05, Welch’s t-test, S19B-D Fig). These results indicate that, under the various exposure protocols used, after 5 days of cultivation no significant phototoxicity resulting in changes in the biofilm biomass, fluorescence intensity, or live cell count were observed.

References

1. Sun W, Shi S, Chen J, Zhao W, Chen T, Li G, et al. Microbiol Spectr. 2022 Sep 27;10(5):e02460-22.

Section 4: Technical details and design considerations

Imaging interval and well position alignment details

Our tests showed good accuracy of the imaging interval and the movement of the sample tray (Fig 4) under the imaging conditions used (shutter speed, resolution, image storage method, etc.). However, if those parameters are changed, the accuracy of the imaging time interval may be affected. When the camera resolution was increased, image acquisition required more resources which affected the reproducibility of the imaging interval. Performance will vary with component specifications

The more significant deviations in sample positioning visible in S13 Fig for later time points are likely the results of friction and we have been able to minimize these with further lubrication of actuators.

Resolution discussion

Although the measured system resolution of 0.14 mm is limited (S3 and S4 Figs) it is not a major issue since the system was developed to capture macroscopic features (ranging from 1 mm to 25 mm in scale) of bacterial aggregates like biofilms and colonies. The camera used has a maximum image resolution of 9248 x 6944 pixels (64 MP), which is comparable or higher than cameras used with common microscopes. However, magnification lenses can’t be readily mounted on this model. The use of other cameras such as the Pi HQ that can be fitted with magnifying lenses could solve this issue and allow for higher magnification when desirable.

LED output specifications

In Fig 5, we confirmed the suitability of the illumination module and optical filter using a strain that constitutively expresses fluorescent proteins. However, genes with lower expression levels may require different conditions. If the exposure intensity of the illumination module used in this experiment is insufficient, a more powerful LED could be used. Refining the spectrum of the illumination LEDs could be done by simply adding appropriate filters in front of the array.

Controller specifications

Our current system is not well-suited for continuous high-resolution imaging (above 2312 × 1736 pixels) or for long-term image analysis over several days. However, by employing a higher-spec single-board computer such as the Jetson Orin Nano Super Developer Kit (https://www.nvidia.com/en-us/autonomous-machines/embedded-systems/jetson-orin/nano-super-developer-kit), it would be feasible to achieve automatic alignment after image analysis as well as continuous high-resolution image acquisition.

Noise level and S/N ratio

Detailed noise measurements and signal-to-noise ratio calculations are presented in S16 and S17 Figs. The noise level was 1.0 or less at all measurement points in the green and red fluorescence images. Signal-to-noise ratios (S/N) exceeded 3 at 380 min in all replicates with noise levels remaining stable (green fluorescence mode CV = 8.03%, red fluorescence mode CV = 8.52%) throughout 5 days of imaging.

Fluorescence inhomogeneity technical explanations

Fluorescent intensity measured with fluorescent disks was reproducible over time and in different directions of analysis (Fig 6). However, greater variation was observed for the red alignment disk. The effect could be attributed to the weaker red fluorescence signals resulting from emission filter characteristics as well as image acquisition settings. Additionally, a glass-like circular reflection in the shape of the alignment disk observed only in red fluorescence mode may influence the profile but is unlikely to occur in bacterial biofilms or other thin biological samples and thus should not be a significant concern.

Hardware degradation

Components most likely to degrade are the LED and the stepping motor. However, after six months of continuous operation, no acute alignment problems caused by the stepping motor were observed. As shown in Fig 6C-F and S11 Fig, the fluorescence intensity of the alignment disk decreased with continued use, a sign of LED wear. This is a common problem that should not affect significantly relative comparisons. Absolute intensity changes might be suppressed by changing the resistance connected to the LEDs or adapting a cooling mechanism to mitigate LED heat generation. The 3D printed components, all made of

PLA [1], should not be affected by heat at the low temperatures (< 57°C). Heat generation by the LEDs or stepping motors used is low and no significant effects on LOTUS' frame were observed.

1. https://3d-printmaster.de/mediafiles/Bilder/Creality-Ender-Plus-PLA-TDS.pdf

Customizability and adaptation to different sample formats (well plates)

LOTUS is designed for macro-scale imaging (1 mm to 25 mm). System customizability is based on the premise of macro-scale observation and includes the ability to extend the actuator rails, add or modify the sample tray to accommodate multi-well plates, and change the LED boards and optical filters for other specific applications. To implement further magnification physical space for mounting lenses on the Pi camera may require significant modifications to the basic scaffold.

As examples, of customizability, we modified the shape of the sample tray to accommodate a 96-well plate for multimode imaging (S6 Fig). The only changes made were to the shape of the sample stage and the alignment position of the wells. Therefore, alignment can be adjusted to accommodate 6, 12, 24, and 48 wells. However, reducing the diameter of the wells may cause light leakage to adjacent wells, and phototoxicity or fluorescence bleaching. Therefore, it is desirable to adjust the size of the holes in the separator, to match the shape of the wells.

As for throughput, it can be increased by expanding the sample tray size to accommodate 12 or more samples, or dishes of various diameters while considering incubator internal dimensions. In addition, increasing the number of samples will result in longer imaging cycles. This issue and the ability of imaging at shorter time intervals could be resolved using high-torque stepping motors.

Section 5: Measurement considerations and interpretations

Comments on biomass measurements and normalization using transillumination

Although our biomass quantification using transillumination cannot distinguish cellular from extracellular contributions, previous studies have shown that extracellular matrix often comprises 70-95% of biofilm dry mass. While this suggests that in many cases the majority of biofilm biomass is not accounted for by cells, under given environmental conditions, this proportion is relatively stable, so variations in transparency should approximate total biomass (cell + matrix) changes within about a two-fold uncertainty.

The simplicity of optical transparency measurements makes it well-suited for screening of multiple samples in a relative manner and avoids the complexity of using double fluorescent reporters such as promoter strength, chromophore maturation, pH/O_2 dependence, spectral bleed, etc. We therefore consider this a robust approach for estimating total biomass. However, significant changes in matrix or extracellular polymeric substances contribution to signal intensity could affect the results. Additional validation under specific cases could further clarify how proportionality is affected under specific experimental conditions.

Comments about fluorescence measurements

In this study, we do not claim that the observed GFP fluorescence levels directly or accurately reflect absolute intracellular gene expression or protein abundance. In the absence of direct validation (e.g., Western blot, etc.), we consider fluorescence intensity as an indirect but reliable proxy for relative expression levels, consistent with previous studies [1-2]. While GFP fluorescence can be influenced by multiple factors such as protein folding, maturation rate, pH, and oxygen availability, these effects are expected to apply similarly across our experimental conditions. Thus, they should not compromise our relative quantitative comparisons.

In addition, because our main objective is the validation of fluorescent protein measurements (detection and expression level) with LOTUS, we use biomass normalized fluorescence intensity. While this index reflects cumulative expression rather than instantaneous promoter activity, it provides a reliable readout for comparing relative expression levels for LOTUS validation and also report on system stability.

Actual promoter activity can be calculated from the time derivative of normalized fluorescence signals [1-2].

References

1. French S, Coutts BE, Brown ED. Cell Syst. 2018 Sep 26;7(3):339-346.e3.

2. Zaslaver A, Bren A, Ronen M, Itzkovitz S, Kikoin I, Shavit S, et al. Nat Methods. 2006 Aug;3(8):623–8

Sensitivity estimation for detection of GFP expression (fluorescence) in biofilms

To estimate the absolute GFP dynamic range in biofilms using LOTUS we used the following estimates of biofilm volume and cell density within biofilms, combined with reported quantum efficiency and extinction coefficients for fluorescein and GFP and LOTUS LOD for fluorescein.

Biofilm volume:

- Radius: 1 cm
- Thickness: 100 μm = 0.01 cm
- Volume = $\pi \times r^2 \times h = \pi \times 1^2 \times 0.01 = 0.0314$ cm³

LOD for fluorescein (this work): 10^{-7} M (conservative, S6 Fig)

Quantum yield:

- Fluorescein: ~ 0.85-0.95 [1-2]
- GFP: ~ 0.60-0.80 [3]
- Correction factor: GFP is roughly 0.7x less efficient

Extinction coefficients:

- Fluorescein: ~ 80,000 $M^{-1}cm^{-1}$ [1-2]
- GFP: ~ 55,000 $M^{-1}cm^{-1}$ [3]
- Correction factor: GFP displays ~ 0.7x less absorption

Overall: GFP is roughly 0.5x as bright as fluorescein

Effective GFP detection limit: 10^{-7} M fluorescein equivalent ÷ 0.5 = 2×10^{-7} M GFP equivalent

Minimum detectable GFP per biofilm:

2×10^{-7} M × 0.0314 cm³ × 6.02×10^{23} molecules/mol × 10^{-3} L/cm³ = 3.8×10^{12} GFP molecules total

Biofilm cell density estimates:

Typical *E. coli* culture density: 10^9 cells/mL (10^9 cells/cm³) [4].

Biofilm densities may thus vary 10^8-10^{11} cells/cm³ depending on maturation and conditions

- Mature bacterial biofilms: 10^{10}-10^{11} cells/cm³ [5]
- Highly packed regions: up to 10^{11} cells/cm³
- Conservative estimate: 10^{10} cells/cm³

Total cells in biofilm: cell density x biofilm volume = $10^{10} \times 0.0314$ **=** 3.14×10^8 cells

Per-cell detection limit:

3.8×10^{12} molecules ÷ 3.14×10^{8} cells = ~12,000 GFP (1.2×10^{4}) molecules per cell

Previous studies of absolute protein abundance in *E. coli* have shown that typical proteins are present at 10^{2}-10^{3} copies per cell, while abundant ones can reach 10^{5}-10^{6} copies per cell [6]. Depending on translation efficiency, GFP absolute abundance may exceed these values. From these results, we estimate that LOTUS can detect:

- High expressers: 10^{6}-10^{7} GFP molecules/cell (clear)
- Moderate expressers: 10^{4}-10^{5} GFP molecules/cell (good)
- Some low to moderate expressers: 10^{2}-10^{4} GFP molecules/cell (marginal ability)

This estimate suggests LOTUS is suitable for screening and detection of moderately expressed genes in *E. coli*. However, readers should keep in mind that this is provided only as a rough, back-of-the-envelope estimate.

References

1. Magde D, Wong R, Seybold PG. Photochemistry and Photobiology 2002;75(4):327–34.
2. Seybold PG, Gouterman M, Callis J. Photochemistry and Photobiology 1969 ;9(3):229–42.
3. Patterson GH, Knobel SM, Sharif WD, Kain SR, Piston DW. Biophys J. 1997 Nov;73(5):2782–90.
4. Milo R, Phillips R, Cell Biology by the Numbers. Garland Science, 2015
5. Hou J, et al. Sci Rep 2019 July 5;9(1):9794.
6. Csibra E, Stan GB. Nat Commun 2022 Nov 3 ;13(1):6600.

Section 6: Comparison with other imaging systems

We have not performed direct comparative benchmarking for other open-source systems. However, here is a summary comparison of features with five similar open-source DIY systems:

Source	Features	References
€100 Lab	Microscopy-focused, single sample	[1]
Picroscope	24-well but single illumination mode	[2]
Futo et al.	Eliminates condensation but opens plates (contamination risk)	[3]
PFIbox	Single fluorescence mode, no filter exchange	[4]
Nuñez et al.	Multi-color fluorescence, no bright-field or filter exchange	[5]
OpenIVIS	Multiple wells but no automated filter switching	[6]

LOTUS advantages: Combines multi-mode illumination, automatic filter exchange, and multi-sample automation in single open-source platform

The open-source system FlyPi was designed for microscopic observation and is capable of bright-field and fluorescence observation using RGB LEDs and UV LEDs [1]. However, to image multiple samples simultaneously, multiple imaging systems should be used, considerably increasing costs. In contrast, LOTUS implements an actuator to move samples, enabling observation of multiple samples using a single observation system.

Picroscope is capable of imaging multiple samples with multiple cameras and capture images from a total of 24 wells simultaneously [2]. However, it is limited to a single illumination mode (white light only), restricting observations to bright-field mode. In comparison, LOTUS allows for flexible customization of light sources and filters according to application needs, thereby enabling fluorescence imaging.

Futo *et al*. developed a system that eliminates image blurring caused by condensation by opening and closing the lid of a Petri dish with a robotic arm [3]. However, this poses a contamination risk, necessitating the installation of a sterilization light and a humidity control mechanism to regulate water evaporation from the agar. On the other hand, with LOTUS, under the conditions used, condensation was not observed.

Other systems capable of performing time-lapse observation of colonies in fluorescence mode have been described [4-5]. Using a single blue transilluminator and an amber filter, enabled temporal observation of green as well as orange/red fluorescence. However, since the optical filters cannot be exchanged, accurate fluorescence quantification may be less reliable and bright-field illumination is absent. By contrast, LOTUS allows for customization of the light source and filters according to the application, enabling observation of both bright-field and fluorescence.

Finally, OpenIVIS is an imaging system capable of capturing multiple wells in a single observation [6]. This system uses RGB LEDs as a light source, but it lacks a function to automatically change optical filters, resulting in low selectivity with multiple fluorescent probes. LOTUS enables customization of light sources and filters according to experimental needs, allowing acquisition of both bright-field and fluorescence images.

As for commercially available imaging solutions, they offer reliable and finely-tuned components and some accommodate a wide variety of sample, imaging modes, and also include specific software for image analysis. However, their costs scale rapidly with feature number and performance limiting their continuous use for many laboratories. In comparison LOTUS uses inexpensive LEDs, stepping motors, a single-board computer and a frame made with a 3D printer. Additionally, the system's customizability and low cost of repairs is another significant advantage over commercially available systems.

Example commercial system with similar features: Celloger Nano

https://midsci.com/item/ASCELLOGNANO/Curiosis-reg-Celloger-Nano/

References

1. Chagas AM, Prieto-Godino LL, Arrenberg AB, Baden T. PLOS Biol. 2017 Jul 18;15(7): e2002702.
2. Ly VT, Baudin PV, Pansodtee P, Jung EA, Voitiuk K, Rosen YM, et al. Commun Biol. 2021 Nov 4;4:1261.
3. Futo M, Široki T, Koska S, Čorak N, Tušar A, Domazet-Lošo M, et al. Sci Rep. 2022 Dec 7;12(1): 21120.
4. French S, Coutts BE, Brown ED. Cell Syst. 2018 Sep 26;7(3):339-346.e3.
5. Nuñez I, Matute T, Herrera R, Keymer J, Marzullo T, Rudge T, et al. PLoS ONE. 2017 Nov 15;12

(11):e0187163.

6. Branning Jr. JM, Faughnan KA, Tomson AA, Bell GJ, Isbell SM, DeGroot A, et al. PLOS ONE. 2024 Mar 18;19(3):e0299875.

Section 7: Data availability

The following are available on our github repository: https://github.com/mrobert7/MSB_imaging

- Python and ImageJ macro scripts
- STL files for 3D printing
- Complete assembly instructions and wiring diagrams and videos

Raw data available upon request to robert.martin.4m “at” kyoto-u.ac.jp
